# Dynamic Single-Atom Catalysts on Gallium To Overcome the Scaling Relationship Limit: AIMD Screening for CO_2_ Reduction and Hydrogen Evolution Reactions

**DOI:** 10.1021/jacsau.5c00823

**Published:** 2025-08-20

**Authors:** Mohsen Tamtaji, William A. Goddard, Ziyang Hu, Shuguang Chen, GuanHua Chen

**Affiliations:** † 645827 Hong Kong Quantum AI Lab Limited, Pak Shek Kok, Hong Kong SAR 999077, China; ‡ Materials and Process Simulation Center (MSC), MC 139-74, 6469 California Institute of Technology, Pasadena, California 91125, United States; § Department of Chemistry, 25809 The University of Hong Kong, Pokfulam Road, Pok Fu Lam, Hong Kong SAR 999077, China

**Keywords:** formic acid, methanol, methane, DFT, overpotential, dephasing function

## Abstract

Extensive research has been conducted on single-atom catalysts (SACs) for a range of electrochemical reactions. However, *static* SACs suffer from scaling relationship limits, which hinder their further development. In this work, we introduce the idea of *dynamic* SACs supported on Gallium for the hydrogen evolution reaction (HER) and the CO_2_ reduction reaction (CO_2_RR). We utilized AIMD and DFT calculations to systematically conduct high-throughput screening on s-, p-, d-, and f-block elements supported by Gallium denoted as M-SAC@Ga. We found that among all the understudied catalysts, Re-, Pt-, Pd-, Rh-, Ir-, Au-, Ag-, Ru-, Tc-, Ni-, Cu-, Os-, Hg-, and Ge-SAC@Ga possess thermodynamic and electrochemical stabilities. In addition: Ni-SAC@Ga leads to CO_2_RR overpotentials of 0.28, 0.28, 0.69, and 0.92 V, respectively, toward CHOOH, CO, CH_3_OH, and CH_4_ formation. Low overpotentials and mitigation of scaling relationship limits are primarily due to the atomic intelligence (the ability to guide reactions) and dynamic coordination changes of SACs, seen through DFT and AIMD calculations. Analyzing the phonon-induced fluctuations in total energies suggests a standard deviation of up to 0.26 V in the calculated overpotentials. Additionally, the dephasing time for these dynamic systems is below 5 fs, a crucial factor affecting the modeling of catalytic behavior. Feature importance analysis suggests that the d-electron numbers serve as the universal descriptors for these catalysts. This study offers a comprehensive insight into the discovery of cutting-edge electrocatalysts and beyond by applying the concept of *dynamic* SACs.

## Graphical abstract

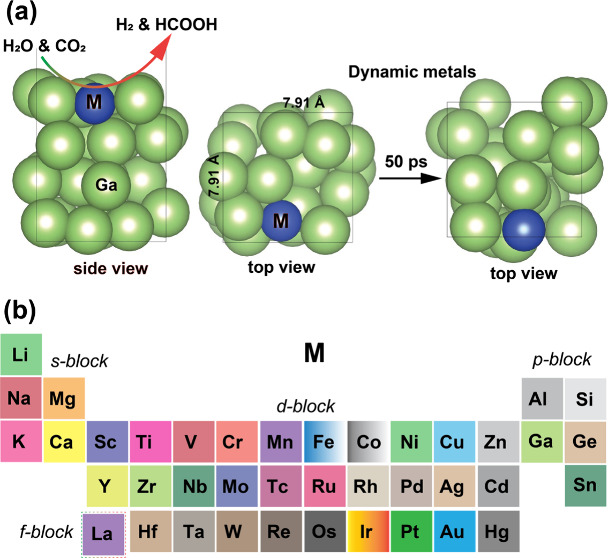

## Introduction

With fossil fuel reliance contributing to a 1.1 °C rise in global temperatures since 1850 and projected increases of up to 3 °C by midcentury,^1,2^ the development of efficient, durable, and affordable catalysts is critical to curbing greenhouse gas emissions and stabilizing ecosystems disrupted by extreme weather and rising heat levels. The hydrogen evolution reaction (HER) can be used in fuel cells for energy storage applications, and the CO_2_ reduction reaction (CO_2_RR) can generate value-added products^3–5^ at room temperature for energy conversion applications to support the ambitious target of net-zero emissions by 2050 ^2^ and mitigate climate change. Single-, dual-, and multiatom catalysts (SACs, DACs, and MACs) are widely reported theoretically and applied experimentally for energy storage and conversion applications.^6^ For instance, a FeCoNiRu-SAC was explored theoretically for CO_2_RR and HER toward syngas production.^7^ The coordination environment of such SACs and DACs is nondynamic; therefore, altering their electrocatalytic activity requires changing the coordination environment during the synthesis process itself. This can lead to the scaling relationship limit and impede the development of advanced electrocatalysts.^7^ Consequently, to address the limitations posed by the nondynamic environment surrounding the active site, we have utilized *dynamic* SACs supported on liquid gallium (Ga).

Liquid metals such as gallium, mercury, cesium, and rubidium are fascinating due to their unique properties, including low melting points and supercooling, positioning them as promising candidates for sustainable and efficient catalytic processes.^8,9^ Liquid metals can rearrange interfacial atom positions in response to surface processes to guide and drive reactions, known as atomic intelligence, where atoms in a freely moving liquid state are directed into specific locations for enhanced functionality. This property provides energetically favorable reaction pathways, allowing reactions that cannot occur on solid-state catalysts.^10^ Their extensive applications have recently resulted in the application of transition metal-doped liquid metals in various chemical reactions, including electrocatalysis, photocatalysis, thermocatalysis, and mechanocatalysis.^11–16^ For example, a Cu-doped gallium liquid metal was reported theoretically and synthesized experimentally for ammonia synthesis, taking advantage of the metal mobility.^17^ In another work, Sn- and Ni-doped gallium liquid metal was reported theoretically and experimentally for the selective synthesis of propylene (C_3_H_6_) from various precursors such as decane.^18^ The mobility of Sn and Ni dopants on the surface of liquid gallium led to precise configurations of reactants and intermediates, leading to mobility-induced activity enhancement. In addition, atomically dispersed Pt metal into the liquid gallium was reported for the oxidation of CH_3_OH at low temperature^19^

The atomic motion in liquid metals, when used as a support for SACs, may provide a unique opportunity to modify the dopant’s coordination environment and enhance their catalytic activity and selectivity. These advantages position liquid metals as an innovative way to design *dynamic* SACs. Consequently, the strategic design of *dynamic* SACs requires in-depth and systematic electrochemical examinations. As far as we know, no *dynamic* SAC on liquid gallium has yet been systematically examined for HER or CO_2_RR.

In this work, we use liquid Ga metal for the CO_2_RR and HER through high-throughput computational screening over 39 different elements containing s-, p-, d-, and f-block elements. We discovered that Re-, Pt-, Pd-, Rh-, Ir-, Au-, Ag-, Ru-, Tc-, Ni-, Cu-, Os-, Hg-, and Ge-SAC@Ga possess thermodynamic and electrochemical stabilities and are appropriate for the HER and CO_2_RR. The dynamic behavior of Ni single atoms is ultimately investigated in the presence and absence of reaction intermediates, and the dephasing time for these systems is calculated to indicate the role of quantum coherence.

Our study pioneers the concept of *dynamic* SACs on liquid gallium, marking a paradigm shift from conventional *static* SACs, to mitigate climate change and support the net-zero emission goal by 2050. The dynamic coordination environment, enabled by the intrinsic atomic mobility and atomic intelligence behavior of liquid metals, allows us to break the traditional scaling relationship limit (*R* ^2^ < 0.3653), a milestone unattainable with conventional SACs. To address the limitations of DFT calculations at 0 K in capturing realistic catalytic dynamics, we employed high-throughput AIMD simulations at 300 K. These simulations revealed the impact of thermal fluctuations on the standard deviation of obtained overpotentials, offering a more accurate representation of catalytic behavior under operational conditions. Furthermore, we introduce the concept of the dephasing function to quantify coherence and extract dephasing times in *dynamic* catalytic systems, an essential parameter influencing catalytic performance. Ultimately, these innovations redefine the principles of catalyst design, offering unprecedented adaptability and mechanistic insight into real-time catalytic processes, providing a foundational understanding for the next generation of *dynamic* catalysts and dynamic behavior on the surface of *static* catalysts in electrochemistry and beyond.

## Materials and Methods

### Ab Initio Molecular Dynamics (AIMD) and Density Functional Theory (DFT) Details

Vienna ab initio Simulation Package (VASP 6.1.0) is used to perform AIMD and DFT calculations with Perdew–Burke–Ernzerhof (PBE) functional.^20,21^ We used the VASPsol code (implicit solvation)^23^ with a dielectric constant of 78.4 for water to include the solvation corrections.^24^ The D3 Becke-Johnson damping function is used for van der Waals (London dispersion) interactions.^25^ Due to the limitations of generalized gradient approximation (GGA)-PBE functional in accurately predicting the Gibbs free energies of carbon-containing intermediates, we applied an energy correction of 0.15 eV per C=O,^24^ e.g., for COOH and CHO. In Figure 1a, we illustrate the construction of a box with fixed lattice parameters of 7.91 Å × 7.91 Å, which contains 25 Ga atoms along with a single atom from each of 39 different elements. A 20.7 Å was applied along the *z* direction to remove interactions between periodic images.^26^ A 520 eV plane wave energy cutoff is applied, and the Brillouin zone is sampled by applying the 2 × 2 × 1 Monkhorst–Pack k-point scheme. The energy and force convergence criteria were set to 10^–6^ eV and 0.03 eV Å^–1^, respectively. DFT calculations were used to calculate the formation energies and dissolution potentials to investigate thermodynamic and electrochemical stabilities. To capture the dynamic nature of both reaction intermediates and the catalyst surface, we have used AIMD simulations at 300 K using the canonical (NVT) ensemble, which maintains a constant volume and temperature. Inspired by the recent paper,^27^ the simulation was run for 25,000 steps with 2 fs timesteps, leading to 50 ps. We believe that 50 ps is adequate for each reaction intermediate to be stabilized on the catalyst surface and reach its lowest energy level, with fluctuations occurring due to the continuous dynamic nature of the liquid Ga catalyst surface and reaction intermediates. Therefore, to calculate Gibbs free energies, we have averaged the energy levels from 40 to 50 ps, during which the system reaches the lowest energy level. For Density of States (DOS) calculations, we used the energy convergence criterion of 10^–7^ eV and the 4 × 4 × 1 Monkhorst–Pack k-point scheme for the Brillouin Zone. VESTA and VASPKIT were used to visualize and postprocess AIMD results, respectively. Python was used to automatically create the input structures, run the calculations, and read and analyze the AIMD and DFT results.

**Figure 1:**
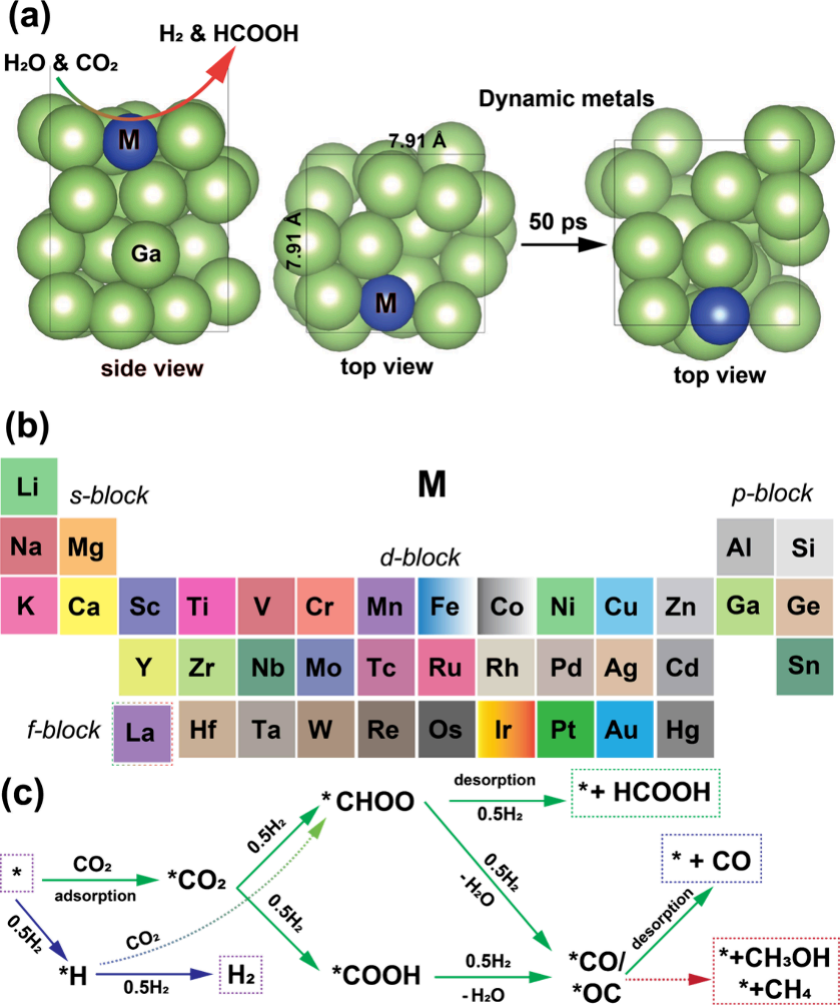
Dynamic single-atom catalysts (SACs). (a) Structure of M-SAC@Ga, in which M represents s-, d-, p-, and f-block elements. Green and blue balls represent Ga and M elements, respectively. (b) s-, d-, p-, and f-block elements used as *dynamic* SACs supported on liquid gallium. (c) Overview of HER and CO_2_RR mechanisms on M-SAC@Ga toward H_2_, CHOOH, CO, CH_3_OH, and CH_4_ formation.

## Results and Discussion

Doping various elements into a liquid metal, such as gallium, brings about the opportunity to dynamically change the coordination environment of active elements in order to enhance their electrocatalytic activity. The structure of doped liquid gallium (M-SAC@Ga), where M represents s-, p-, d-, and f-block elements, is depicted in Figure 1a,b. By using AIMD calculations, we discovered that the coordination environment of single-atom catalysts (SACs) changes dynamically at room temperature (Figure 1a). This is because using a liquid metal like gallium as the support exhibits interatomic motions even at room temperature due to its low melting point of 29.76 °C.^28^

### Stability Analysis

The thermodynamic and electrochemical stabilities of M-SAC@Ga are investigated to assess the dopants’ potential toward aggregation and their dissolution behavior. This analysis is crucial to determine whether the dopants tend to aggregate on the gallium support or undergo dissolution into the electrolyte. The thermodynamic stability was examined based on the formation energy (*E*_formation_) calculation as follows:^29^

$$E_{\mathrm{formation}}=E_{M-\mathrm{SAC}@\mathrm{Ga}}-E_{\mathrm{Ga}}-E_{M}^{\mathrm{bulk}}$$ 1

Here, *E*_M–SAC@Ga_ and *E*_Ga_ are the DFT-calculated total energy of liquid gallium with and without the dopants (M), and *E*_M_^bulk^ denotes the energy of the elements in their most stable bulk structure (see Table S2). We used the dissociation potential (*U*_diss_, V) to examine the electrochemical stability of M-SAC@Ga:^29^

$$U_{\mathrm{diss}}=U_{\mathrm{diss}}^{o}-\frac{E_{\mathrm{formation}}}{Ne}$$ 2

where *U*_diss_^o^ and *N* correspond to the standard dissolution potential of elements in their bulk structure and the number of electrons involved in the dissolution process, respectively (see Table S2). Table S2 shows the values for the dissolution potentials (*U*_dis_, V) and formation energies (*E*_formation_, eV), and Figure 2a displays the dissolution potential (*U*_dis_, V) against the formation energy (*E*_formation_, eV) for all elements. Figure 2a indicates the electrochemical and thermodynamic stabilities of Re-, Pt-, Pd-, Rh-, Ir-, Au-, Ag-, Ru-, Tc-, Ni-, Cu-, Os-, Hg-, and Ge-SAC@Ga, based on their positive dissolution potentials and negative formation energies. These 14 elements, primarily transition metals with Ge as an exception, exhibit stable and atomically dispersed structures. Their stability suggests they are unlikely to agglomerate or dissolve into the solution under the negative applied potentials typically used for HER and CO_2_RR. Among them, Re demonstrates the highest thermodynamic stability, while Pt shows the highest electrochemical stability. In contrast, according to Figure 2a and Table S2, W, Cr, and Si exhibit positive formation energies, indicating a tendency to agglomerate on the Ga support. Although s-block elements such as Li, Na, Mg, K, and Ca possess negative formation energies, suggesting thermodynamic stability, their dissolution potentials are highly negative (close to −2 V). This implies that they are unstable under the working potentials used for CO_2_RR and HER. These s-block elements are commonly present in the electrolyte (e.g., KOH) and typically remain dissolved above the applied potential of −2 V. In this regime, they interact near the catalyst surface, participating in proton transfer and influencing reaction kinetics, as discussed in recent papers.^30–32^ However, at potentials more negative than −2 V, these elements may deposit on the surface and potentially function as SACs.

**Figure 2:**
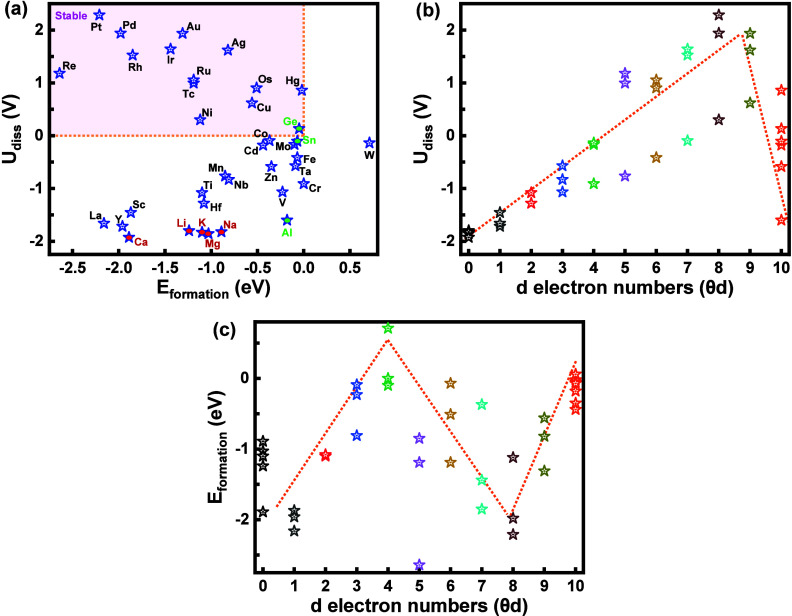
Thermodynamic and electrochemical stability analysis. (a) DFT-calculated dissolution potentials versus formation energies (*E*_formation_) of dopant elements on gallium support. (b) Dissolution potential versus the d-electron numbers (θ_d_) of doped elements. (c) Formation energy versus θ_d_ of doped elements.

Early transition metals such as Sc, Ti, V, Cr, Y, Zr, Nb, Mo, Hf, and Ta; other transition metals, including Mn, Fe, Co, Zn, and Cd; the f-block element La; and p-block elements such as Al and Sn exhibit thermodynamic stability with negative formation energies. However, they are electrochemically unstable due to their negative dissolution potentials, indicating that these elements are unlikely to remain stabilized on the Ga support and are prone to dissolution into the electrolyte.

To further understand the behavior of dopants, we investigated the relationship between the dissolution potential/formation energy and the electronic and atomic properties of the doped elements. Figure 2b,c shows the variation of dissolution potentials and formation energies as a function of the number of d-electrons (θ_d_). A volcano-type trend is observed for the dissolution potential, with elements having θ_d_ values of 7, 8, and 9 exhibiting the highest dissolution potentials and, thus, the greatest electrochemical stability. In contrast, the most negative formation energies, indicating the highest thermodynamic stability, are found for elements with θ_d_ values of 0, 1, and 8. Elements with θ_d_ = 4 show the least thermodynamic stability, as reflected by their most positive formation energies.

Figure S1a,b further explores the correlation between the dissolution potential and (i) Pauling electronegativity and (ii) atomic radius. As electronegativity increases, the dissolution potential becomes more positive, indicating improved electrochemical stability. In contrast, an increase in atomic radius correlates with more negative dissolution potentials, suggesting reduced electrochemical stability. This trend is particularly evident for s-block elements, which have both low electronegativity and large atomic radii, resulting in the most negative dissolution potentials.

To evaluate the impact of reaction intermediates on the thermodynamic and electrochemical stabilities of dopants, we calculated the formation energies and dissolution potentials in the presence of the CHOO intermediate as a representative reaction intermediate. Figure S2a presents the dissolution potentials plotted against formation energies for various dopants in the presence of the CHOO intermediate. Figure S2b compares the formation energies with and without the CHOO intermediate, while Figure S2c shows the corresponding comparison for the dissolution potentials. These results reveal that although Hg and Ge were previously considered stable catalysts, they become prone to aggregation in the presence of the CHOO intermediate. This is evidenced by their formation energies shifting to positive values, indicating a loss of thermodynamic stability under reaction conditions involving the CHOO intermediate. Therefore, it can be concluded that p-block elements are not thermodynamically or electrochemically stable, either in the presence or absence of reaction intermediates, and are prone to aggregation, forming clusters or dissolving into the solution rather than remaining atomically dispersed on the gallium support. This observation aligns with a recent DFT and AIMD simulation study on Bi-doped gallium, as a p-block element, which demonstrated Bi’s tendency to aggregate,^26^ further supporting the instability of p-block elements in single-atom configurations. In contrast, only a select group of transition metals, such as Re, Pt, Pd, Rh, Ir, Au, Ag, Ru, Tc, Ni, Cu, and Os, exhibit both thermodynamic and electrochemical stability in the absence and presence of reaction intermediates, making them ideal candidates for stable SACs on gallium.

To evaluate the oxidation tendency of the catalysts, we calculated the free energy of oxygen, as illustrated in Figure S10. The resulting volcano plot reveals that the strongest oxygen adsorption occurs at a d-electron number of θ_d_ = 5. This indicates that all thermodynamically and electrochemically stable dopants exhibit positive oxygen-free energy.

### Gibbs Free Energy Calculations

Figure 1c shows the overview of HER and CO_2_RR mechanisms on M-SAC@Ga toward H_2_, CHOOH, CO, CH_3_OH, and CH_4_ formation. To evaluate the electrochemical activity of M-SAC@Ga and calculate the thermodynamic overpotentials, we obtained the Gibbs free energy (Δ*G*) of intermediates such as H, COO, CHOO, COOH, CO, OC, COH, and CHO as follows:^24^

$$\Delta G_{\mathrm{int}.}=F_{\mathrm{int}.@M-\mathrm{SAC}@\mathrm{Ga}}-F_{M-\mathrm{SAC}@\mathrm{Ga}}-N_{{\mathrm{CO}}_{2}}F_{{\mathrm{CO}}_{2}}-N_{H_{2}}F_{H_{2}}+N_{H_{2}O}F_{H_{2}O}+\Delta \mathrm{ZPE}-T\Delta S+\int_{0}^{298}C_{V}dT$$ 3

where *F*_int.@M–SAC@Ga_ and *F*_M–SAC@Ga_ rgy of M-SAC@Ga at room temperature over 40 to 50 ps of AIMD simulation in the presence and absence of the intermediate (int.), respectively. *F*_CO_2__, *F*_H_2_O_, and *F*_H_2__ represent the total energy of CO_2_ (g), H_2_O (g), and H_2_ (g), respectively. Zero-point energy (ΔZPE), entropy (*T*Δ*S*), and integrated specific heat (∫_0_^298^ *C*_V_d*T*) are presented in Table S1. N_CO_2__, N_H_2_O_, and N_H_2__ represent the stoichiometric quantities of CO_2_ (g), H_2_O (g), and H_2_ (g), respectively. We also assume that the Gibbs free energy is independent of the pH and linearly correlated with the applied potential (Δ*G*_int.*_(*U*) = Δ*G*_int.*_(*U* = 0) + *N*e*U*), where *N* is the number of electrons transferred.

Figure 3 displays the Gibbs free energy of the H, COO, CHOO, COOH, CO, OC, COH, and CHO intermediates for M-SAC@Ga. The hatched areas indicate that we have not calculated the Gibbs free energy of COH and CHO for those catalysts because they are not selective toward CO_2_RR. Figure S8a shows the Gibbs free energy variation for each intermediate reaction through a box plot, indicating their average and standard deviation. The COOH and CO have larger standard deviations of 0.49 and 0.79 eV, respectively. Contradictory, the COO, as the more neutral intermediate, possesses a lower standard deviation of 0.21 eV and, therefore, a smaller change in the Gibbs free energy. COO intermediate mostly does not form chemical bonds to the M active site and, therefore, is unaffected by changes in the M active site. Examining the COOH and CHOO intermediates reveals that the standard deviation of CHOO is lower than that of COOH. This is because the COOH intermediate forms one bond between its carbon atom and the M active site, whereas the CHOO forms two bonds between its oxygen atoms and the M and Ga active sites. Figure S8b displays a Gibbs free energy variation histogram for 240 data points with an average of 0.78 eV, a skewness of 0.09, and a standard deviation of 0.67 eV.

**Figure 3:**
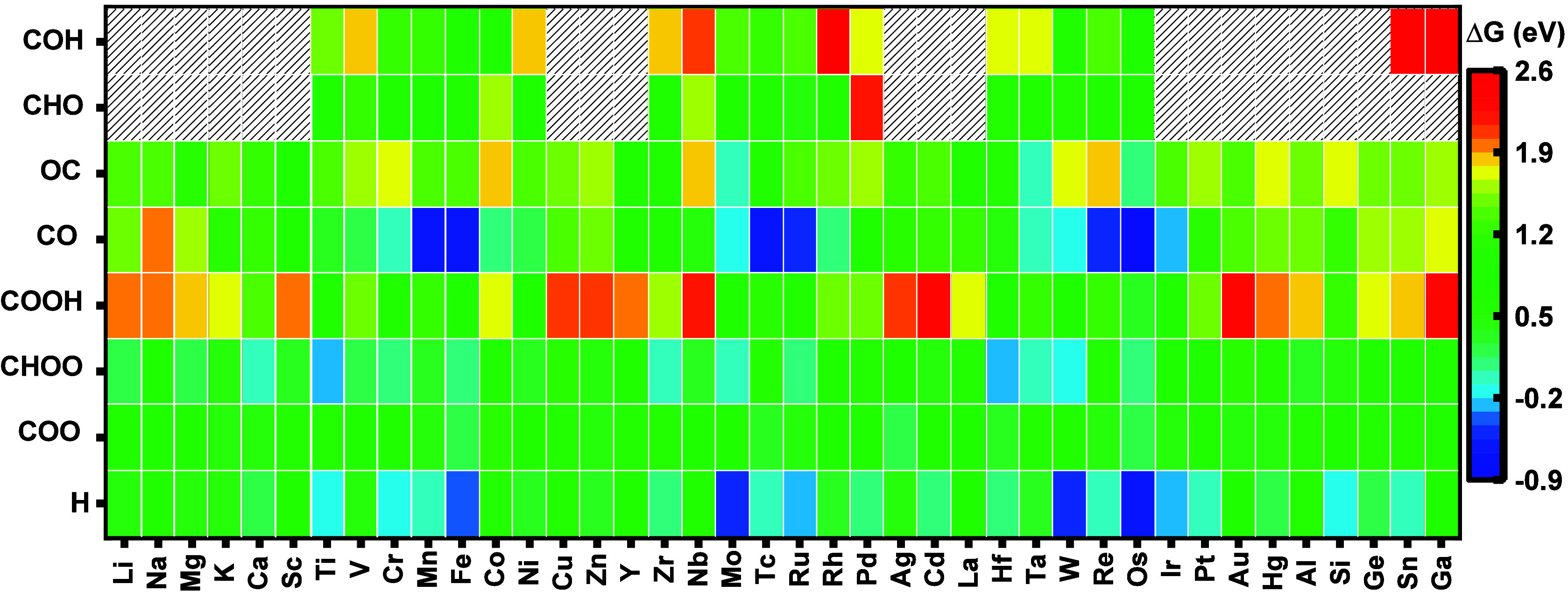
High-throughput screening. AIMD-calculated Gibbs free energy (Δ*G*) of H, COO, CHOO, COOH, CO, OC, COH, and CHO intermediates for M-SAC@Ga.

To study the scaling relationship limit, a hurdle in designing and discovering state-of-the-art electrocatalysts,^35^ we performed a linear regression for the Δ*G*_H_, Δ*G*_COO_, Δ*G*_CHOO_, Δ*G*_COOH_, Δ*G*_CO_, Δ*G*_OC_, Δ*G*_COH_, and Δ*G*_CHO_. When multiple catalytic intermediates are involved, scaling relationships between these intermediates can determine the optimal catalyst. For the HER with only one intermediate, there appears to be no scaling relationship to address. However, it has been established that HER competes with the CO_2_RR, necessitating the tuning of hydrogen adsorption in relation to CHOO or COOH intermediates. Additionally, it has been demonstrated that adsorbed hydrogen on the Cu(100) surface, rather than the hydrogen from water, can participate in CHOO formation.^36^ Therefore, we believe that investigating the scaling relationship with hydrogen is crucial for the CO_2_RR.

Figure 4a,b shows that the scaling relationship limit is broken among the intermediates, aligning with a previous report.^37,38^ Figure 4c,d presents the linear relationship coefficients, *R* ^2^ uncertainties, and *p*-value between the Δ*G*_H_, Δ*G*_COO_, Δ*G*_COOH_, Δ*G*_CO_, Δ*G*_OC,_ Δ*G*_COH_, and Δ*G*_CHO_ and Δ*G*_CHOO_. The low *p*-value indicates that the fitting is statistically significant. The *R* ^2^ values for H, COO, COOH, CO, OC, COH, and CHO intermediates are 0.2448, 0.0367, 0.3410, 0.1586, 0.1727, 0.0876, and 0.3653, respectively. All *R* ^2^ values are below 0.3653, showing a broken linear scaling relationship compared to *static* single- and multiatom catalysts, which have *R* ^2^ values above 0.90, as previously reported.^20^ This is in accordance with our recent paper showing that the *dynamic* SACs within Catenene metal complexes lead to a weakened scaling relationship limit with *R* ^2^ values below 0.84.^29^ In addition, this is in accordance with another report mentioning that Catenane organocatalysts can overcome the scaling relationship limit due to the dynamic motion of the macromolecules, leading to dynamic coordination environments around the active site.^37^ We also performed DFT calculations at 0 K to calculate Gibbs free energies of the reaction intermediates (Figure S3) and evaluate their scaling relationship (Figures S4). The results reveal broken linear scaling, even using static DFT calculations. This suggests that the absence of scaling relations is not solely due to dynamic effects captured by AIMD, but rather may reflect the intrinsic atomic mobility in liquid metals, referred to as atomic intelligence,^10^ being partially captured even using static DFT calculations. To highlight Ga’s inherent atomic mobility, Figure S5c,d shows considerable shifts in atomic positions, obtained from DFT calculations, for Ni-SAC@Ga in the absence and presence of CHOO intermediate. Additionally, Figure S5a,b shows a weak linear correlation in static DFT results (*R* ^2^ = 0.6810), which disappears in AIMD simulations (*R* ^2^ = 0.0474), confirming that AIMD better captures dynamic atomic behavior and its impact on free energy calculations. Additionally, Figure S5a,b demonstrates that the broken scaling relationship enables independent tuning of Δ*G*_CHO_ energies, decoupled from Δ*G*_COH_, which facilitates selective catalyst design. These findings reinforce the unique catalytic potential of SAC-doped liquid gallium systems.

**Figure 4:**
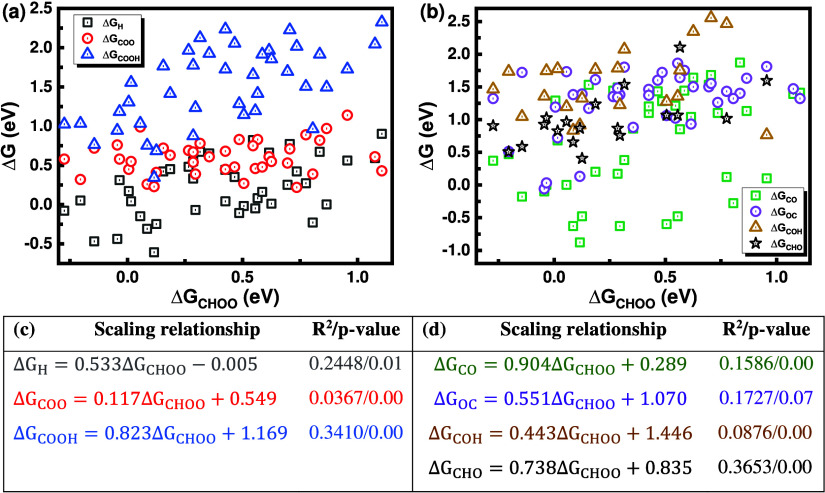
Scaling relationship limits among the intermediates. (a, b) AIMD-calculated Δ*G*_H_, Δ*G*_COO_, Δ*G*_COOH_, Δ*G*_CO_, Δ*G*_OC,_ Δ*G*_COH_, and Δ*G*_CHO_ versus Δ*G*_CHOO_, showing the broken scaling relationship limits. (c, d) Linear relationship coefficients, *R* ^2^, and *p*-value between Δ*G*_H_, Δ*G*_COO_, Δ*G*_COOH_, Δ*G*_CO_, Δ*G*_OC,_ Δ*G*_COH_, and Δ*G*_CHO_ with Δ*G*_CHOO_.

This weakened scaling relationship does not prevent us from achieving a descriptor to predict the catalytic activity of *dynamic* SACs. As depicted in Figure 5a, the relationship of the Gibbs free energy of intermediates versus the d-electron number of active sites shows a strong correlation through a volcano plot with the strongest adsorption energies at the d-electron number of θ_d_ = 6. It is worth mentioning that, because the CHOO intermediate makes two bonds between its oxygen elements with the M and Ga sites of M-SAC@Ga (e.g., see Figure S36), we consider the average of d-electron numbers of M and Ga atoms (θ_d_ = [θ_d,M_ + θ_d,Ga_]/2 in which θ_d,Ga_ = 10). Since the H, COOH, and CO intermediates make only one bond with the M active site, we consider the d-electron number of the M active site (θ_d_ = θ_d,M_). For nonbonding intermediates like COO and OC, no correlation with d-electron number was observed (Figure S9). Therefore, the inclusion of dopants’ or Ga’s d-electron number is not universal but context-dependent, used only when the intermediate interacts with dopants or Ga atoms. This strong dependency of Gibbs free energies on the d-electron numbers is in accordance with our feature importance analysis obtained from machine learning implementation (see section S2 of the Supporting Information file for model details, along with *R* ^2^ and RMSE values). As depicted in Figure 5b, |θ_d_-6| ranks among the highest in both permutation and SHAP feature importance, with a strong effect on the Gibbs free energies as the model output. Therefore, due to the high permutation and SHAP feature importance along with high mutual dependency and Pearson coefficient, we have used |θ_d_-6| as a universal descriptor to define the Gibbs free energy of the CO_2_RR and HER intermediates on s-, p-, d-, and f-block elements supported by gallium. The high mutual dependency indicates that changes in |θ_d_-6| are strongly associated with variations in the Gibbs free energy across different intermediates and catalyst systems. This means that the descriptor consistently influences the model output, regardless of the specific reaction pathway or dopant. The high Pearson coefficient further confirms a strong linear correlation between |θ_d_-6| and the calculated Gibbs free energies.

**Figure 5:**
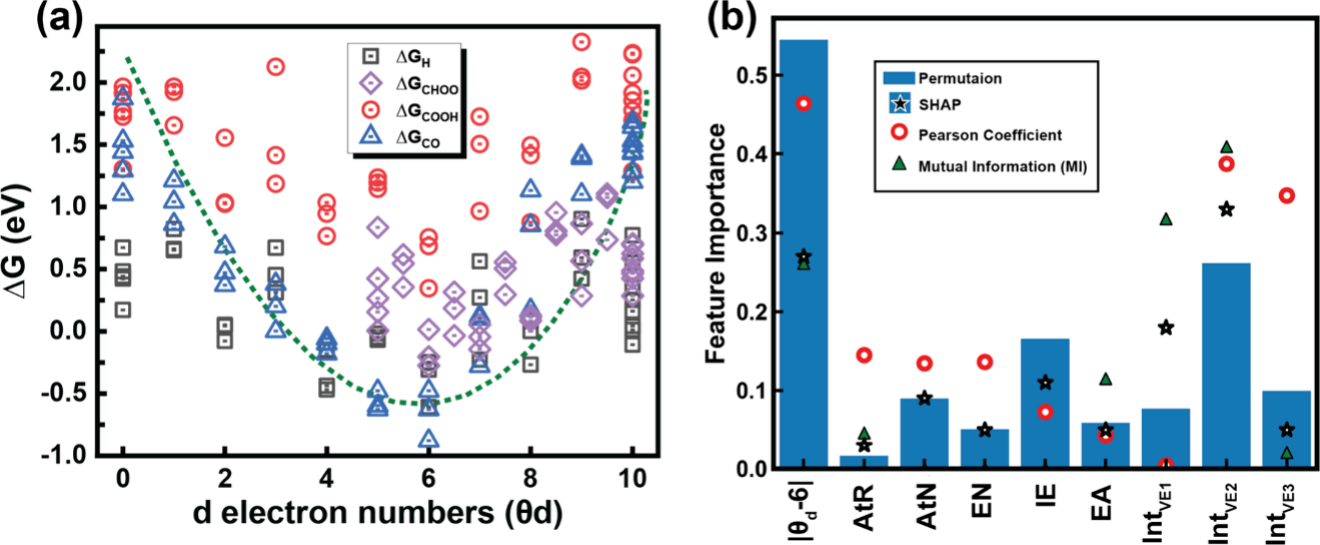
Descriptor. (a) Relationship of Gibbs free energy of intermediates versus the d-electron number (θ_d_) of active sites indicates the strongest adsorption at θ_d_ = 6. (b) Permutation and SHAP feature importance analysis on Δ*G* of reaction intermediates, along with the corresponding Pearson correlation coefficients and Mutual Information (MI). This indicates that |θ_d_-6| is among the most important parameters.

After this in-depth discussion on the Gibbs free energy of HER and CO_2_RR intermediates, we evaluated the CO_2_RR and HER performances of M-SAC@Ga.

### HER

We studied the HER on M-SAC@Ga through the following fundamental step shown in Figure 1c:

$$*+0.5H_{2}⇌H^{*}+e^{-}$$ 4

Figure 6a displays HER profiles for M-SAC@Ga, showing that HER on Pd-, Pt-, Tc-, and Re-SAC@Ga leads to overpotentials of η^HER^ = |Δ*G*_H_|/e = 0.08, 0.01, 0.02, and 0.05 V_RHE_, respectively. This is much better than the HER overpotential of 0.77 V for undoped gallium. Besides, this is comparable to the DFT-obtained overpotentials of 0.01 for Pt-SAC inside the van der Waals (vdW) gap of SnS_2_ ^39^ and the overpotential of 0.10 V within the vdW gap of axially bonded V2N8 dual-atom catalyst.^40^ Figure 6b shows HER overpotentials versus the d-electron numbers (θ_d_) of dopants. Among the stable candidates, such as Re-, Pt-, Pd-, Rh-, Ir-, Au-, Ag-, Ru-, Tc-, Ni-, Cu-, Os-, Hg-, and Ge-SAC@Ga, all candidates except for Au-SAC@Ga exhibit lower and more favorable HER overpotentials compared to undoped gallium. Figure 6c,d displays the partial density of states (PDOS) of 5d_*x*2‑*y*2_, 5d_*z*2_, 5d_*xz*_, 5d_*xy*_, and 5d_*yz*_ orbitals of the Pt site, along with the 1s orbital of the H intermediate in the absence or presence of the H intermediate. We see that the 5d orbitals of the Pt site form bonding orbitals (σ) at *E*–*E*_f_ = −4 to −5.5 eV with the 1s orbital of the H intermediate. The insets show the top view of Pt-SAC@Ga before and after the adsorption of the H intermediate, showing that the atomic distribution of Pt and its coordination environment change during the Pt absorption. The inset also shows the side view of the Bader charge transfer from Pt-SAC@Ga to the H intermediate (isosurface value = 0.002 e/Å^3^). The blue and yellow colors show the regions of charge deficiency and charge availability, respectively.

**Figure 6:**
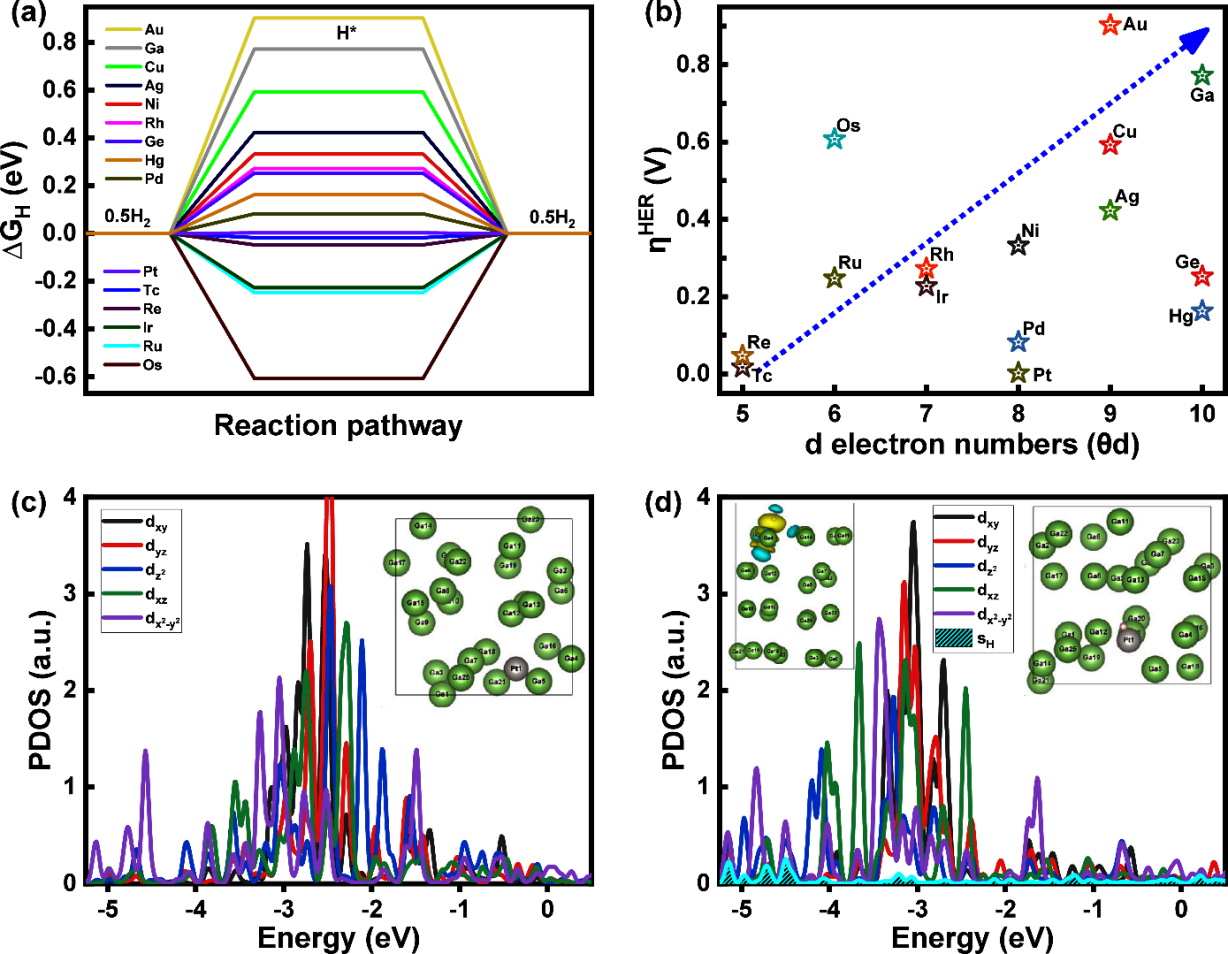
Hydrogen evolution reaction (HER). (a) HER pathways for M-SAC@Ga. (b) HER overpotential versus the d-electron numbers (θ_d_). Partial density of states (PDOS) of 5d_*x*2‑*y*2_, 5d_*z*2_, 5d_*xz*_, 5d_*xy*_, and 5d_*yz*_ orbitals of the Pt active site (c) in the absence and (d) in the presence of the H intermediate. The insets show top views of Pt-SAC@Ga and the side view of the Bader charge transfer from Pt-SAC@Ga to the H intermediate (isosurface value = 0.002 e/Å^3^). The blue and yellow colors show the region of charge deficiency and charge availability, respectively.

### CO_2_ Reduction Reaction (CO_2_RR)

We mainly focused on the 2-electron transfer CO_2_RR toward formic acid (HCOOH) production at room temperature on M-SAC@Ga through the fundamental steps shown in Figure 1c:^41^

$$*+{\mathrm{CO}}_{2}+H^{+}+e^{-}⇌{\mathrm{CHOO}}^{*}$$ 5

$${\mathrm{CHOO}}^{*}+H^{+}+e^{-}⇌*+\mathrm{HCOOH}$$ 6

Before studying the CO_2_RR mechanism, the competitive HER must be discussed. To achieve high selectivity in the CO_2_RR, a catalyst that binds hydrogen weakly but has a strong affinity for CHOO/COOH intermediates might be effective. Figure 7a displays Δ*G*_CHOO_ against Δ*G*_H_, indicating that Ni-SAC@G and undoped gallium show selective CO_2_RR over HER. Although Re-, Pt-, Pd-, Rh-, Ir-, Au-, Ag-, Ru-, Tc-, Cu-, Os-, Hg-, and Ge-SAC@Ga seem HER selective, we have considered an alternative reaction pathway through that the initial proton transfer to CO_2_ can originate from adsorbed hydrogen rather than hydrogen from surrounding water on M-SAC@Ga.^36^ The fundamental steps of this process are illustrated in Figure 1c and as below:

$$*+0.5H_{2}⇌H^{*}+e^{-}$$ 7

$$H^{*}+{\mathrm{CO}}_{2}⇌{\mathrm{CHOO}}^{*}$$ 8

**Figure 7:**
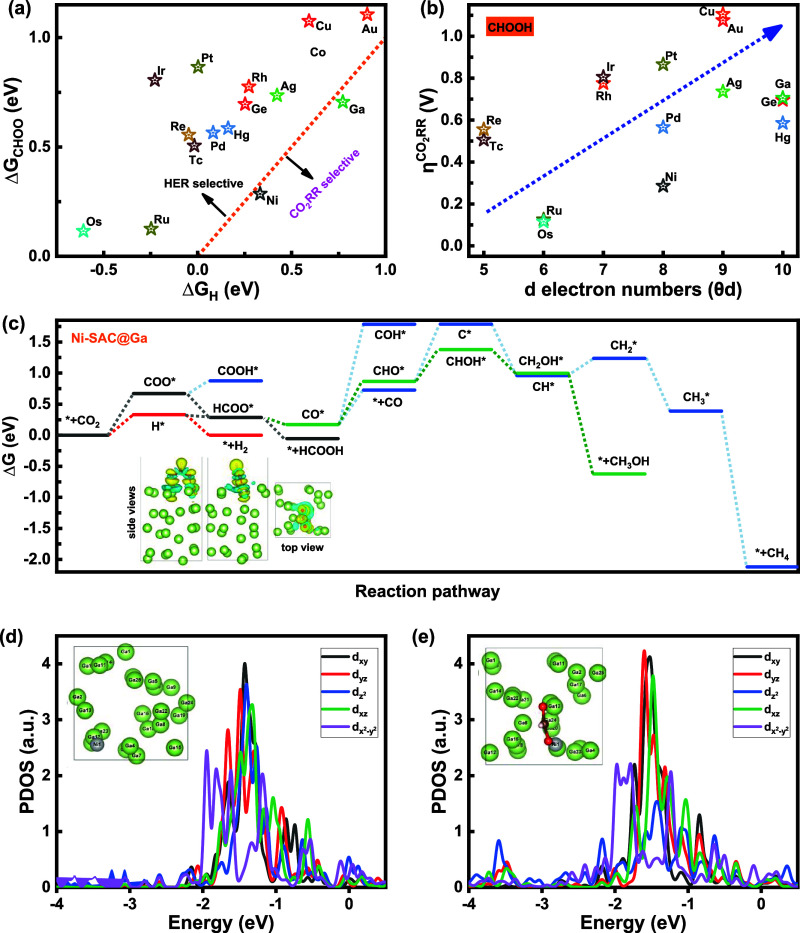
CO_2_RR pathway toward CHOOH, CO, CH_3_OH, and CH_4_ formation. (a) Gibbs free energy of CHOO intermediate (Δ*G*_CHOO_) versus Δ*G*_H_ for M-SAC@Ga. (b) Overpotential of CO_2_RR toward CHOOH versus the d-electron numbers (θ_d_), showing that Os-, Ru-, and Ni-SAC@Ga possess the lowest overpotentials. (c) CO_2_RR pathway for Ni-SAC@Ga with CO_2_RR overpotentials of 0.28, 0.28, 0.69, and 0.92 V_RHE_ toward CHOOH, CO, CH_3_OH, and CH_4_, respectively. The insets display the side views of the Bader charge transfer from Ni-SAC@Ga to CHOO (isosurface value = 0.002 e/Å^3^). The blue and yellow colors show the region of charge deficiency and charge availability, respectively. Partial density of states (PDOS) of 3d_*x*2‑*y*2_, 3d_*z*2_, 3d_*xz*_, 3d_*xy*_, and 3d_*yz*_ orbitals of the Ni active site in Ni-SAC@Ga (d) in the absence and (e) in the presence of the CHOO intermediate.

This implies that Re-, Pt-, Pd-, Rh-, Ir-, Au-, Ag-, Ru-, Tc-, Cu-, Os-, Hg-, and Ge-SAC@Ga can also go through this reaction mechanism toward the formation of a CHOO* intermediate.

Figure 7b shows the CO_2_RR overpotential (toward CHOOH formation) versus d-electron numbers, indicating that Os-, Ru-, Ni-, Tc-, Re-, Pd-, Hg-, and Ge-SAC@Ga possess overpotentials of 0.12, 0.13, 0.28, 0.51, 0.56, 0.57, 0.59, and 0.70 V_RHE_, respectively, lower than the overpotential of 0.71 V for undoped Ga. In contrast, other stable candidates such as Ag-, Rh-, Ir-, Pt-, Cu-, and Au-SAC@Ga show higher overpotentials of 0.74, 0.78, 0.81, 0.87, 1.08, and 1.11 V_RHE_, respectively, than undoped gallium.

In addition to investigating HCOOH production, we also explored the reaction pathways leading to the formation of CO, CH_3_OH, and CH_4_ through the electrochemical steps detailed in the Supporting Information file. Figure S12a illustrates the CO desorption energy versus θ_d_. Pd-, Pt-, Cu-, Au-, Ag-, Ge-, and Hg-SAC@Ga, along with undoped Gallium (−0.95 eV), exhibit exothermic CO desorption of −0.12, −0.40, −0.66, −0.68, −0.37, −0.80, and −0.71 eV, respectively, indicating spontaneous CO production and release, while CH_3_OH and CH_4_ formation remain thermodynamically unfavorable. Figure S12b–d illustrates the CO_2_RR overpotential for CO, CH_3_OH, and CH_4_ formation versus θ_d_. The reaction pathways toward CO production are detailed in Figures S13–S20. Among these, Pd-SAC@Ga demonstrates the lowest CO_2_RR overpotential of 0.57 V toward CO production with * + CO_2_ + H^+^ + e^–^ → CHOO* as the potential determining step (PDS) and the CO desorption energy of −0.12 eV. The reaction pathways of other stable candidates such as Ni-, Rh-, Ru-, Ir-, Re-, Tc-, and Os-SAC@Ga toward H_2_, HCOOH, and CH_3_OH/CH_4_ production are shown in Figures 7c and S21–S26. They show endothermic CO desorption, favoring the protonation of the CO intermediate before desorption, and thus are more likely to facilitate CH_3_OH or CH_4_ formation. Among them, Ni-SAC@Ga shows one of the lowest overpotentials toward CH_3_OH and CH_4_ formation.

Figure 7c displays the CO_2_RR pathway for Ni-SAC@Ga toward CHOOH, CH3OH, and CH4. * + CO_2_ + H^+^ + e^–^ → CHOO* is the PDS with the overpotential of 0.28 V toward CHOOH production. CO* + H^+^ + e^–^ → CHO* is the PDS with the overpotential of 0.69 V toward CH_3_OH production. CHO* + H^+^ + e^–^ → H_2_O + C* is the PDS with the overpotential of 0.92 V toward CH_4_ production. The insets display the side and top views of the Bader charge transfer from Ni-SAC@Ga to the CHOO intermediate (isosurface value = 0.002 e/Å^3^). The blue and yellow colors show the region of charge deficiency and charge availability, respectively. Figure 7d,e displays the PDOS of 3d_*x*2‑*y*2_, 3d_*z*2_, 3d_*xz*_, 3d_*xy*_, and 3d_*yz*_ orbitals of the Ni active site in Ni-SAC@Ga in the absence and presence of the CHOO intermediate. Figure S27 displays the PDOS of the p_*z*_, p_*x*_, and p_*y*_ orbitals of the O atom in the CHOO intermediate that is adsorbed on Ni-SAC@Ga. We see that the 3d_*z*2_ and 3d_*yz*_ orbitals of the Ni active metal site form bonding orbitals (σ) at *E*–*E*_f_ = −3.0 to −4.0 eV with the p_*z*_, p_*x*_, and p_*y*_ orbitals of the O atom in the CHOO intermediate. The insets show the top view of Ni-SAC@Ga before and after the adsorption of the CHOO intermediate, showing that the atomic distribution of Ni and its coordination environment change during the time.

To consider the effect of the reaction kinetics, we investigated the energy barriers for the formation of CHOOH and CO from the CHOO* intermediate on the Ni-SAC@Ga catalyst. Figure S7 shows the free energy barrier for ${\mathrm{CHOO}}^{*}\overset{H^{+}+e^{-}}{\to }{\mathrm{CHOOH}}^{*}$ and ${\mathrm{CHOO}}^{*}\overset{H^{+}+e^{-}}{\to }{\mathrm{CO}}^{*}+H_{2}O$ . It shows the maximum energy barrier of 0.08 and 0.53 eV for ${\mathrm{CHOO}}^{*}\overset{H^{+}+e^{-}}{\to }{\mathrm{CHOOH}}^{*}$ in the forward and backward directions, respectively. It shows the maximum energy barrier of 0.93 and 0.19 eV for ${\mathrm{CHOO}}^{*}\overset{H^{+}+e^{-}}{\to }{\mathrm{CO}}^{*}+H_{2}O$ in the forward and backward directions, respectively. It suggests that the production of CHOOH is kinetically more favorable than that of CO, on Ni-SAC@Ga. Additional energy barrier calculations considering the effects of explicit solvation, pH, electrolyte composition, and applied potentials are necessary to comprehensively address the reaction kinetics of these processes. However, such analyses fall beyond the scope of this study, which primarily focuses on the reaction mechanism from the thermodynamic point of view rather than from the kinetic point of view.

### Dynamic Behavior Investigation

We investigated the dynamic behavior of M-SAC@Ga catalysts. Figures 8a,b and S29a,b display the mean squared displacement (MSD) of the Ni atom and Ga atoms in Ni-SAC@Ga system in the presence and absence of CHOO intermediate, obtained from the following equation:^42^

$$\mathrm{MSD}(\delta t)=\langle |\vec{r}(\delta t)-\vec{r}(0){|}^{2}\rangle$$ 9

where *r⃗* represents an atom’s position vector, and δ*t* denotes a time step of 2 fs. The insets show the top view of Ni-SAC@Ga in the presence of the CHOO intermediate at 0 and 50 ps. In addition, the Supporting Information Video shows how the interatomic motions change from 0 to 50 ps for Ni-SAC@Ga in the presence of the CHOO intermediate. These show that the atomic distributions of Ga and Ni change over time, providing insight into the dynamic behavior of these understudied catalysts. Examining Figures 8a,b and S29a,b, we observe that without the CHOO intermediate, the MSD of the Ni atom and Ga atoms is primarily along the *y*- and *x*-axis, and the MSD along the *z*-axis is nearly zero. Upon adsorption of the CHOO intermediate, the MSD is suppressed in the *y*- and *x*-axis. This occurs because CHOO forms two bonds with the Ni and Ga active sites along the *y*-axis, restricting Ni and Ga movement in the *x* and *y* directions. Based on Figures 8a,b and S29a,b, we observe that the MSD continues to change and increase even after 50 ps. This indicates that atoms on the surface and bulk are in constant motion, and the catalysts are thermally stable, while the reaction intermediate is stabilized on the surface and reaches its lowest average energy level. As shown in Figure 8c, the energy of the Ni-SAC@Ga system, both in the presence and absence of the intermediate, reaches a minimum after approximately 50 ps of simulation. This suggests that the simulation time is sufficient to capture the system’s minimum energy state. At this energy minimum, the total energy continues to fluctuate due to the dynamic behavior of the reaction intermediates and the liquid Ga catalyst surface. As shown in Figures 8c and S30, this fluctuation leads to the total energy standard deviations of 0.16 and 0.21 eV for Ni-SAC@Ga in the absence and presence of the CHOO intermediate, respectively, showing that after the introduction of the CHOO intermediate on the catalyst surface, the standard deviation in the energy level increases. This results in the standard deviation of $\sqrt{0.16^{2}+0.21^{2}}=0.26$ eV for Δ*G*_CHOO_ and the standard deviation of (0.26 eV)/e = 0.26 V for CO_2_RR overpotential. Therefore, Ni-SAC@Ga possesses the CO_2_RR overpotential of 0.28 V ± 0.26 V. Besides, standard deviations of between 0.10 and 0.31 eV are observed for the Gibbs free energy of intermediates across different elements as SACs. This suggests that the Gibbs free energies and overpotentials achieved in this study always possess an uncertainty. Considering the high standard deviations in Gibbs free energies an overpotentials, we acknowledge the experimental feasibility challenges associated with achieving theoretically predicted low overpotentials. Specifically, while our AIMD simulations indicate that Ni-SAC@Ga can achieve a CO_2_RR overpotential as low as 0.28 V, the phonon-induced energy fluctuations introduce an uncertainty of ±0.26 V. This means that, in practical experimental conditions, the observed overpotential could be between 0.02 and 0.54 V. These fluctuations reflect the dynamic nature of the catalyst surface and intermediates and highlight the importance of accounting for thermal and quantum effects when evaluating catalyst performance.

**Figure 8:**
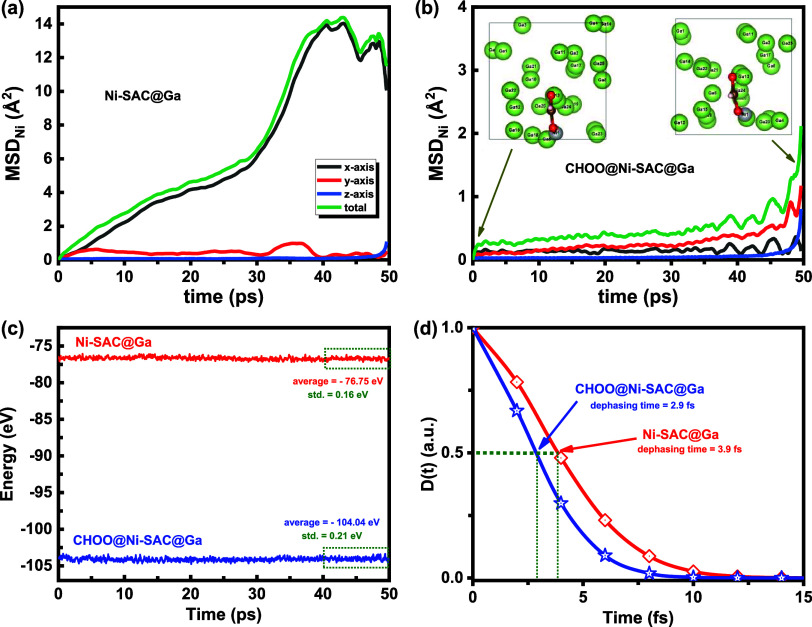
Dynamic behavior. Mean squared displacement (MSD) of Ni atom in Ni-SAC@Ga system (a) in the absence and (b) in the presence of the CHOO intermediate. The insets show the top view of Ni-SAC@Ga in the presence of the CHOO intermediate at 0 and 50 ps. (c) Total energy of Ni-SAC@Ga and CHOO@Ni-SAC@Ga from 0 to 50 ps obtained from AIMD-D3 calculations, indicating the stabilized energy level between 40 and 50 ps. (d) Phonon-induced electronic dephasing (D­(t)) in Ni-SAC@Ga and CHOO@Ni-SAC@Ga, related to the total energy at 300 K from 40 to 50 ps.

Another critical aspect to consider is the behavior of dopants in liquid gallium, specifically whether they remain on the surface or migrate into the bulk. Our study focuses on evaluating the catalytic behavior of thermodynamically and electrochemically stable dopants on the surface of liquid gallium. However, recent literature indicates that certain atoms tend to deplete from the surface and diffuse into the bulk of gallium, while some tend to protrude from the surface. For instance, Bi atoms have been observed to move toward the surface during cooling from 300 to 40 °C.^26^ Additionally, AIMD simulations at 450 K have shown that Bi tends to migrate to the surface, while atoms like Au, Pt, and Sn are more likely to be depleted into the bulk, and Ag and Li tend to be both on the surface and inside the bulk of gallium.^43^ Similar surface depletions have been reported for Pd@Ga using AIMD simulations as well as X-ray photoelectron spectroscopy at 400 to 700 K,^44^ Pd@Ga using AIMD simulations at 320.15 K,^45^ Ni@Ga using AIMD at 900 °C,^46^ and Rh@Ga.^47^ In another study, AIMD simulations at 423.15 K indicate that Sn atoms tend to protrude from the surface, while Ni atoms are depleted from the interface and are present below the interface in the GaSn_0.029_Ni_0.023_ system.^18^ Although the behavior of Sn appears contradictory across different systems such as Sn@Ga and GaSn_0.029_Ni_0.023_,^18,43^ it needs to be indicated that the surface dynamics of dopants are hugely influenced by several factors, including the presence of co-dopants, whether or not the surface is oxidized, and system temperature along with the atomic size, electronegativity, valence, and concentration of dopants.

In our work, we further studied the behavior of Ni in liquid gallium across two different concentrations and temperatures. As shown in Figure S32, we have investigated the dynamic behavior of two Ni atoms on 24 Ga atoms at 300 K using AIMD simulation and found that one of the Ni atoms goes inside the bulk of the gallium, while the other Ni atom resides on the surface of the gallium. In addition, as illustrated in Figure S33, when a single Ni atom is placed on top of 25 Ga atoms, AIMD simulations at 300 K reveal that the Ni atom remains on the surface. However, upon increasing the temperature to 673.15 K, the Ni atom begins to oscillate between the bulk and the surface (Figure S34). This behavior aligns with recent AIMD studies at 673 K, which show that Cu similarly fluctuates between the surface and bulk of gallium.^17^ These findings suggest that single dopant atoms have the potential to dynamically migrate to the surface of liquid gallium and serve as active catalytic sites, a phenomenon reported as an explanation for the superior performance of Supported Catalytically Active Liquid Metal Solutions (SCALMS).^48^ For example, theoretical findings have suggested that although Pt atoms tend to deplete from the surface of Pt@Ga, they can dynamically reappear on the surface, and the adsorption of molecules keeps Pt atoms on the surface.^49^ Similarly, AIMD simulations have demonstrated that Rh tends to migrate away from the interface; however, in the presence of CO, Rh remains on the surface and serves as an active site.^47^ This behavior is attributed to the strong affinity of CO molecules, e.g., an intermediate in our study, for binding to dopant atoms. This interaction can draw the depleted atoms back to the interface, highlighting the dynamic nature of the dopants. At the same time, strong interactions between the reaction intermediates and dopants can lead to adsorbate-induced segregation. To investigate this effect in the Ni-SAC@Ga system, we performed a separate AIMD simulation at 300 K for 50 ps, modeling two Ni atoms on the Ga surface in the presence of the CHOO intermediate. As shown in Figure S35, the distance between the Ni atoms remains greater than the typical Ni–Ni bond length (≃2.5 Å), while both atoms stay on the surface. This indicates that the presence of the CHOO intermediate does not significantly promote the segregation of Ni atoms.

Therefore, although several experimental and AIMD studies have reported the depletion of certain transition metals from the interface, these findings are primarily based on clean surface conditions. We believe that the behavior of the dopants in the presence of reaction intermediates differs significantly. As a result, evaluating the catalytic activity of dopants located on the surface remains a feasible and meaningful approach.

Indeed, studying the full dynamic behavior of dopants on the surface and inside the bulk of gallium is one of the challenging tasks, being affected by various factors, which needs separate and comprehensive studies, which is out of focus of our current study, which focuses on the thermodynamic catalytic activity of thermodynamically and electrochemically stable dopants on the surface of gallium. To extend our findings, we also explored the catalytic activity of Ni-SAC@Ga, where the Ni atom is embedded within the bulk of gallium, as shown in Figure S28. This shows that in the case of Ni in the gallium bulk, * + CO_2_ + H^+^ + e^–^ → CHOO* is the PDS with the overpotential of 0.50 V toward both CHOOH, CO, and CH_4_ formation. While CHO* + H^+^ + e^–^ → CHOH* is the PDS with the overpotential of 0.90 V toward CH_3_OH formation.

We also investigated phonon-induced decoherence in the energy levels using AIMD simulations at 300 K, which is not captured by DFT calculations performed at 0 K. The decoherence in the energy level can be investigated through the dephasing function):^50^

$$D(t,T)=e^{-g(t,T)}$$ 10

The line shape function is:^50^

$$g(t,T)=\frac{1}{\hbar^{2}}\int_{0}^{t}d\tau_{1}\int_{0}^{\tau_{1}}d\tau_{2}C(\tau_{2},T)$$ 11

where *c*(τ_2_,*t*) = ⟨Δ*F*(τ_2_,*t*)­Δ*F*(0,*t*)⟩ and Δ*F*(τ_2_,*t*) = *F*(τ_2_,*t*) – ⟨*F*(τ_2_,*t*)⟩ is the autocorrelation function. Square brackets ⟨···⟩ represents time-averaging and $\hbar =\frac{h}{2\pi }=6.582119569\times 10^{-16}\mathrm{eV}\;s$ is the reduced Planck’s constant. The autocorrelation function and time-dephasing function are obtained for the total energy of Ni-SAC@Ga in the absence and presence of the CHOO intermediate from our AIMD simulations at 300 K from 40 to 50 ps, as shown in Figures S31 and 8d. The dephasing time, at *D*(*t*) = 0.5, decreases from 3.9 to 2.9 fs after the introduction of the CHOO intermediate, suggesting that the quantum coherence loss is quick, below 5 fs, and the surface with the intermediate experiences a quicker quantum coherence loss due to higher energy fluctuations. In DFT calculations, due to the overlooking of energy level fluctuations, *D*(*t*) is always 1, the dephasing time is infinite, and the quantum system is considered coherent. This introduces significant errors in the calculations and justification of the dynamic behavior of the catalyst and intermediates. We anticipate that decoherence effects may also be significant in *static* catalysts and should be carefully considered and justified when evaluating catalyst behavior. The short dephasing times observed in our study suggest that 0 K DFT calculations are only valid over extremely short time scales. Moreover, the rapid loss of quantum coherence implies that electron–phonon coupling is significant and must be considered, particularly for its impact on the charge transfer to the reaction intermediates. To address this, future studies should explicitly calculate dephasing times and investigate how electron–phonon interactions influence the charge transfer kinetics and catalytic performance in electrocatalysis.

## Conclusions

This study provides insights into the performance of *dynamic* SACs for room-temperature electrochemistry. *Static* SACs have been extensively reported for many electrochemical reactions. However, they often suffer from scaling relationship limits. Thus, for the Ga liquid support, we examine *dynamic* SACs with a *dynamic* coordination environment for the HER and the CO_2_RR. Applying AIMD and DFT calculations, we study the thermodynamic and electrochemical stabilities of s-, p-, d-, and f-block elements and the adsorption energies of reaction intermediates. We found that some dopants, such as Pd, Pt, Tc, and Re, enhanced the HER performance, and some dopants, such as Ni, Pd, and Pt, enhanced the CO_2_RR performance. We found that the scaling relationship for SACs is broken in this study. In summary, the extensive AIMD calculations provided in this study lay the groundwork for the community to conduct additional quantum mechanics calculations for *dynamic* single- and dual-atom catalysts supported on gallium or other liquid metals in addition to synthesizing them. This provides the opportunity to use the atomic intelligence of liquid metals to facilitate C–C coupling for CO_2_RR toward C_2+_ products, C–N coupling toward urea production, O–O coupling for OER, and O–O dissociation for ORR.

## Supplementary Material





## Data Availability

Data will be available on request.

## Notes

AIMD results, machine learning details, and additional figures as mentioned in the text (PDF)
Video of CHOO intermediate on Ni-SAC@Ga (MP4)
