# Supplementary material for: Dynamic Single-Atom Catalysts on Gallium To Overcome the Scaling Relationship Limit: AIMD Screening for CO2 Reduction and Hydrogen Evolution Reactions
Source: JACS Au. 2025 Aug 20;5(9):4459–71. doi: 10.1021/jacsau.5c00823 (PMC12458042; doi:10.1021/jacsau.5c00823)
Supplement: Supplementary file 1 [file au5c00823_si_001.pdf]

Supporting Information

Dynamic Single-Atom Catalysts on Gallium to Overcome Scaling Relationship Limit: AIMD Screening for CO_2 Reduction and Hydrogen Evolution Reactions

Mohsen Tamtaji[1], William A. Goddard III[2], Ziyang Hu[1], Shuguang Chen[1], GuanHua Chen[1,3*]*

[1]Hong Kong Quantum AI Lab Limited, Pak Shek Kok, Hong Kong SAR, 999077, China

[2]Materials and Process Simulation Center (MSC), MC 139-74, California Institute of Technology, Pasadena CA, 91125, USA

[3]Department of Chemistry, The University of Hong Kong, Pokfulam Road, Hong Kong SAR, 999077, China

*Corresponding Authors' email: ghc@everest.hku.hk and wag@caltech.edu

S1. DFT and AIMD calculation results

Table S1. Values utilized for entropy (TS) and zero-point energy (ZPE) corrections and integrated specific heat in calculating the free energy of reactants, products, and intermediate species adsorbed on M-SAC@Ga. All energy values are presented in eV.

Species	T×S at 298.15 K	ZPE	C=O correction	$\int C_V dT$
**H***	0.000	0.170	0.00	0.010
**COO***	-0.132	0.310	0.00	0.055
**CHOO***	-0.221	0.530	0.00	0.106
**COOH***	-0.228	0.610	0.15	0.104
**CO***	-0.111	0.220	0.00	0.056
**COH***	-0.106	0.470	0.00	0.057
**CHO***	-0.107	0.450	0.15	0.056
**H_2(g)**	0.410	0.270	---	---
**H_2O(g)**	0.580	0.570	---	---
**CO_2(g)**	0.660	0.350	---	---

Table S2. DFT-calculated formation energies ($E_{formation}$) and dissolution potentials, along with the total energy of the elements in their most stable bulk phase (E_M^{bulk}), the number of electrons transferred during dissolution (*N*), and the standard dissolution potential (U_{diss}^{o}).

**Element**	E_M^{bulk} **(eV)**[1]	*N*[1–5]	U_{diss}^{o} **(V)**[1–5]	$E_{formation}$ **(eV)**	U_{diss} **(V)**
Li	-1.94[a]	1	-3.04	-1.24	-1.80
Na	-1.48[a]	1	-2.71	-0.89	-1.82
Mg	-1.36[a]	2	-2.37	-1.03	-1.86
K	-1.31[a]	1	-2.93	-1.10	-1.83
Ca	-1.81[a]	2	-2.87	-1.89	-1.93
Sc	-6.20	3	-2.08	-1.87	-1.46
Ti	-7.76	2	-1.63	-1.10	-1.08
V	-8.93	2	-1.18	-0.23	-1.07
Cr	-9.49	2	-0.91	0.00	-0.91
Mn	-8.32	2	-1.19	-0.85	-0.77
Fe	-8.45	2	-0.45	-0.07	-0.42
Co	-7.10	2	-0.28	-0.37	-0.10
Ni	-5.55	2	-0.26	-1.12	0.30
Cu	-3.71	2	0.34	-0.56	0.62
Zn	-1.27	2	-0.76	-0.35	-0.59
Y	-6.46	3	-2.37	-1.96	-1.72
Zr	-8.48	4	-1.45	NC	NC
Nb	-10.08	3	-1.10	-0.81	-0.83
Mo	-10.95	3	-0.20	-0.10	-0.17
Tc	-10.30	2	0.40	-1.19	1.00
Ru	-9.20	2	0.46	-1.19	1.06
Rh	-7.27	2	0.60	-1.85	1.52
Pd	-5.17	2	0.95	-1.98	1.94
Ag	-2.76	1	0.80	-0.82	1.62
Cd	-0.90	2	-0.40	-0.44	-0.18
La	-4.93[a]	3	-2.38	-2.16	-1.66
Hf	-9.96	4	-1.55	-1.08	-1.28
Ta	-11.87	3	-0.60	-0.09	-0.57
W	-13.01	3	0.10	0.71	-0.14
Re	-10.05	3	0.30	-2.64	1.18
Os	-11.23	8	0.84	-0.51	0.91
Ir	-8.80	3	1.16	-1.44	1.64
Pt	-6.03	2	1.18	-2.21	2.29
Au	-3.15	3	1.50	-1.31	1.94
Hg	-0.76[a]	2	0.86	-0.02	0.87
Al	-3.75	3	-1.66	-0.18	-1.60
Si	-5.41[a]	---	---	0.06	NC
Ge	-4.52[a]	4	0.10	-0.05	0.12
Sn	-3.85	2	-0.14	-0.07	-0.11
Bi	-3.91	3	0.50	0.13	0.46

[a]: calculated in this work, ---: not available, NC: Not calculated

CO_2RR toward CO on M-SAC@Ga through the following steps:

$$\mathrm{CHOO^* + H^+ + e^- \rightleftharpoons H_2O + CO^*} \tag{S1}$$

$$\mathrm{CO^* \rightleftharpoons * + CO\ (desorption)} \tag{S2}$$

CO_2RR toward CH_3OH on M-SAC@Ga through the following steps:

$$\mathrm{CHOO^* + H^+ + e^- \rightleftharpoons H_2O + CO^*} \tag{S3}$$

$$\mathrm{CO^* + H^+ + e^- \rightleftharpoons CHO^*/COH^*} \tag{S4}$$

$$\mathrm{CHO^*/COH^* + H^+ + e^- \rightleftharpoons CHOH^*} \tag{S5}$$

$$\mathrm{CHOH^* + H^+ + e^- \rightleftharpoons CH_2OH^*} \tag{S6}$$

$$\mathrm{CH_2OH^* + H^+ + e^- \rightleftharpoons * + CH_3OH} \tag{S7}$$

CO_2RR toward CH_4 on M-SAC@Ga through the following steps:

$$\mathrm{CHOO^* + H^+ + e^- \rightleftharpoons H_2O + CO^*} \tag{S8}$$

$$\mathrm{CO^* + H^+ + e^- \rightleftharpoons CHO^*/COH^*} \tag{S9}$$

$$\mathrm{CHO^*/COH^* + H^+ + e^- \rightleftharpoons H_2O + C^*} \tag{S10}$$

$$\mathrm{C^* + H^+ + e^- \rightleftharpoons CH^*} \tag{S11}$$

$$\mathrm{CH^* + H^+ + e^- \rightleftharpoons CH_2^*} \tag{S12}$$

$$\mathrm{CH_2^* + H^+ + e^- \rightleftharpoons CH_3^*} \tag{S13}$$

$$\mathrm{CH_3^* + H^+ + e^- \rightleftharpoons * + CH_4} \tag{S14}$$

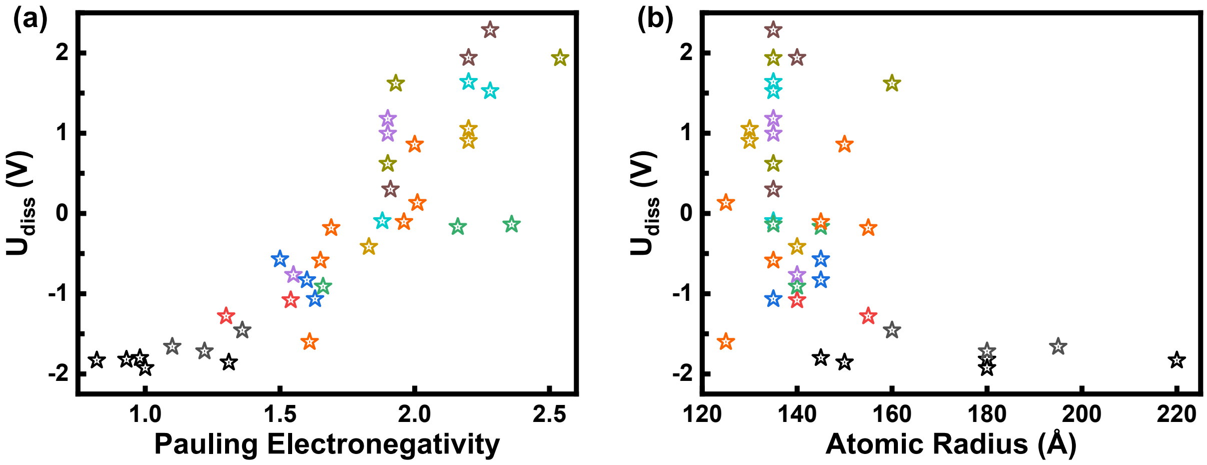

Figure S1. Dissolution potential versus the (a) Pauling electronegativity and (b) empirical atomic radius of doped elements.

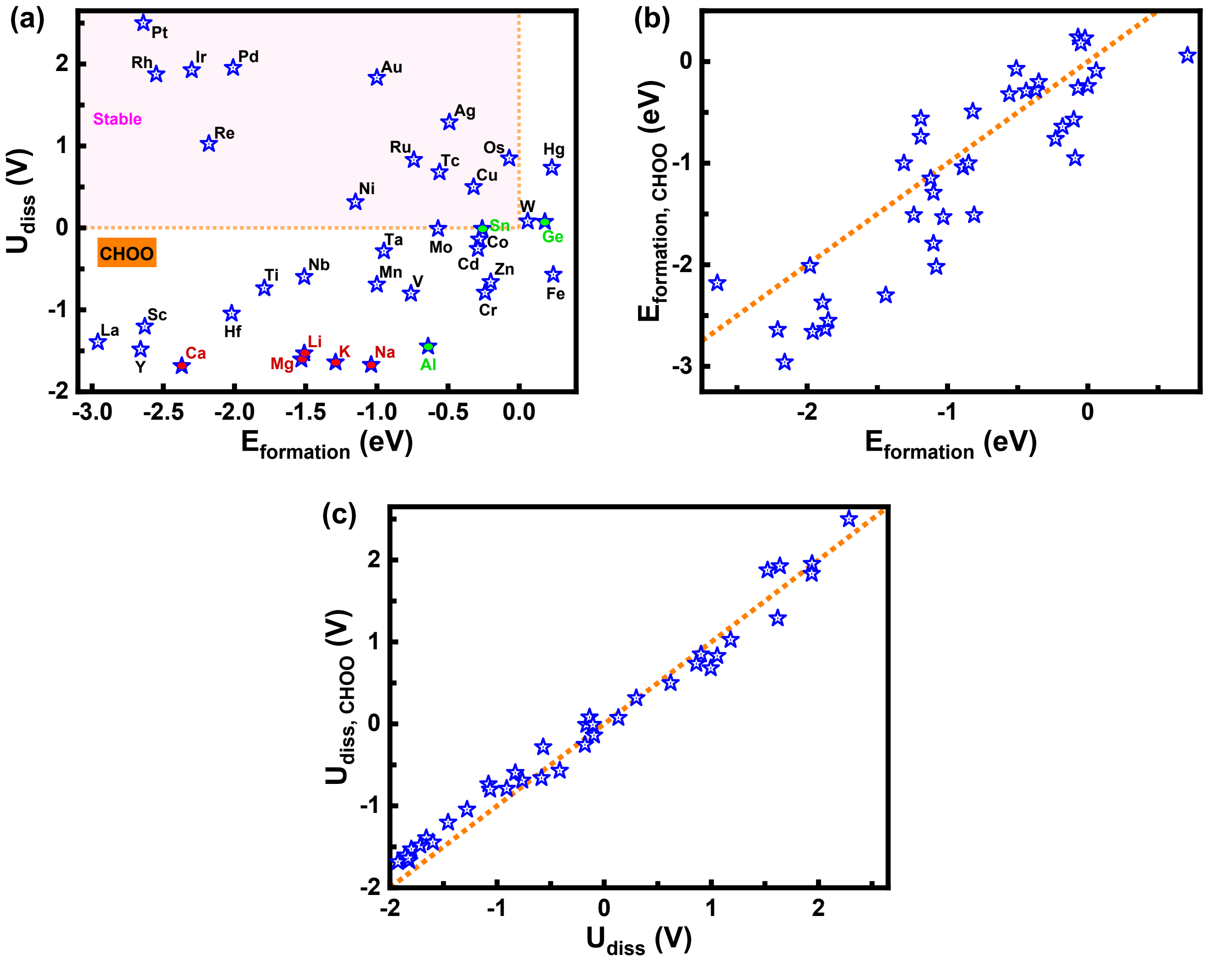

Figure S2. Thermodynamic and Electrochemical Stability Analysis in the Presence of the CHOO Intermediate. (a) DFT-calculated dissolution potentials versus formation energies ($E_{formation}$) of dopant elements on a Gallium support in the presence of the CHOO intermediate. (b) Formation energies of dopants on Gallium support in the presence versus absence of the CHOO intermediate. (c) Dissolution potentials of dopants on Gallium support in the presence versus absence of the CHOO intermediate.

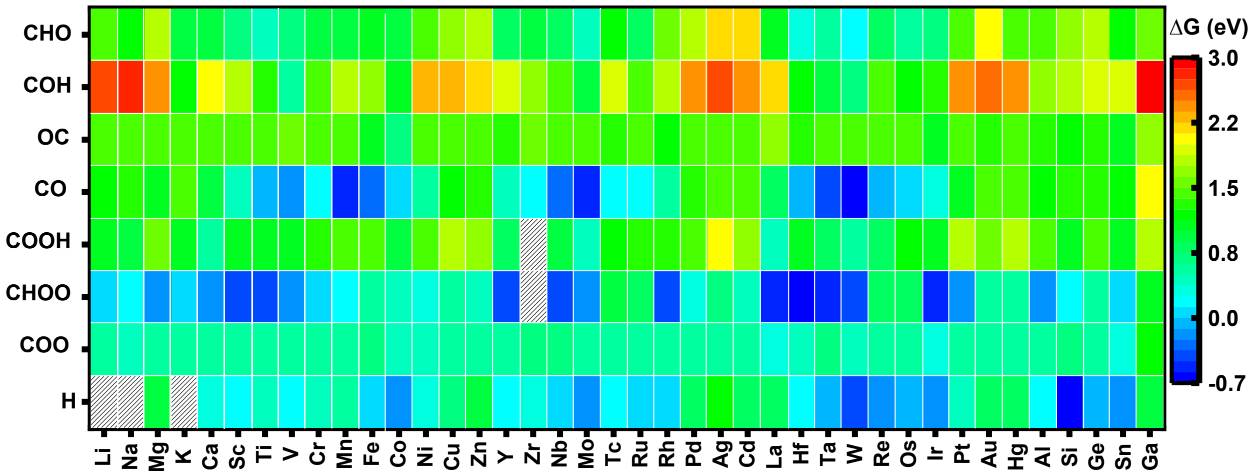

Figure S3. High-throughput screening. DFT-calculated Gibbs free energy (ΔG) of H, COO, CHOO, COOH, CO, OC, COH, and CHO intermediates for M-SAC@Ga.

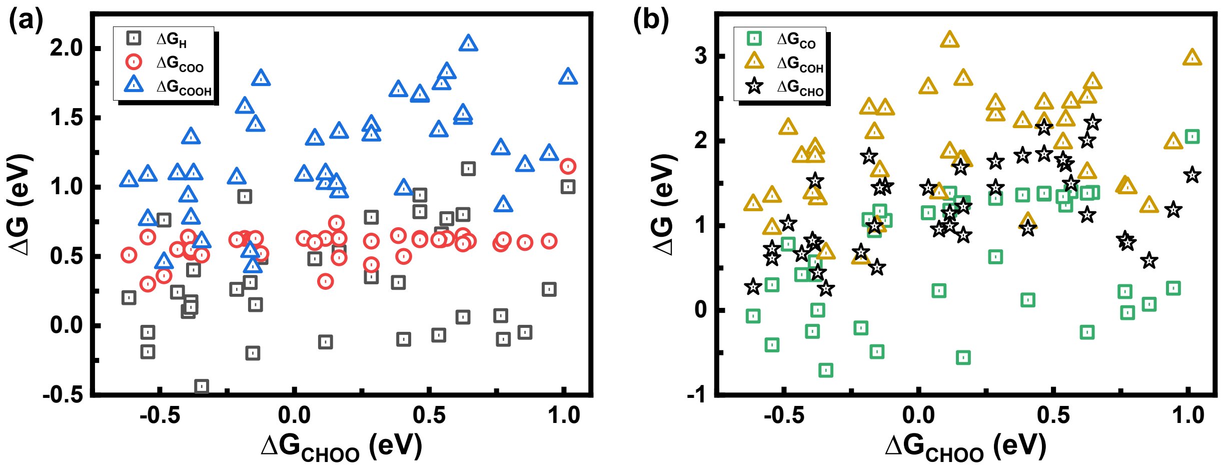

(c) Scaling relationship	R²-value	(d) Scaling relationship	R²-value
$\Delta G_H = 0.227\Delta G_{CHOO} + 0.276$	0.0684	$\Delta G_{CO} = 0.567\Delta G_{CHOO} + 0.567$	0.1373
$\Delta G_{COO} = 0.118\Delta G_{CHOO} + 0.578$	0.1776	$\Delta G_{COH} = 0.500\Delta G_{CHOO} + 1.764$	0.0814
$\Delta G_{COOH} = 0.453\Delta G_{CHOO} + 1.166$	0.2869	$\Delta G_{CHO} = 0.5438\Delta G_{CHOO} + 1.119$	0.2328

Figure S4. Scaling relationship limits among the intermediates. (a,b) DFT-calculated ΔG_H, ΔG_{COO}, ΔG_{COOH}, ΔG_{CO}, ΔG_{COH}, and ΔG_{CHO} versus ΔG_{CHOO}. (c,d) Linear relationship coefficients and R^2 between ΔG_H, ΔG_{COO}, ΔG_{COOH}, ΔG_{CO}, ΔG_{COH}, and ΔG_{CHO} with ΔG_{CHOO}.

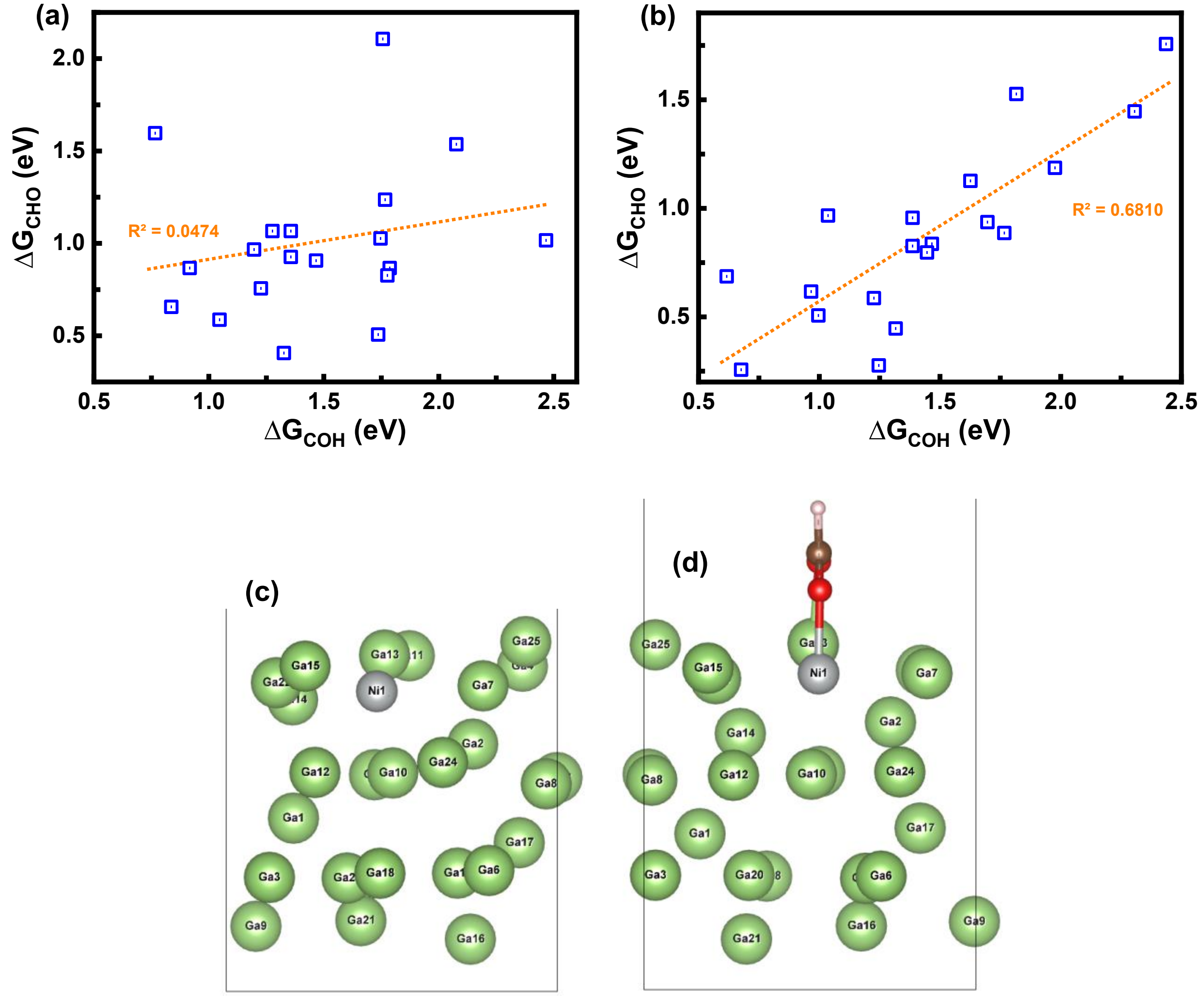

Figure S5. Scaling relationship limits among the intermediates. ΔG_{CHO} versus ΔG_{COH}, based on (a) AIMD calculation and (b) DFT calculation results. Shifts in atomic positions, obtained from DFT calculations, for Ni-SAC@Ga in the (c) absence and (d) presence of CHOO intermediate. Please note that the initial atomic configuration of Ni and Ga atoms (POSCAR files) used for structural relaxation was identical.

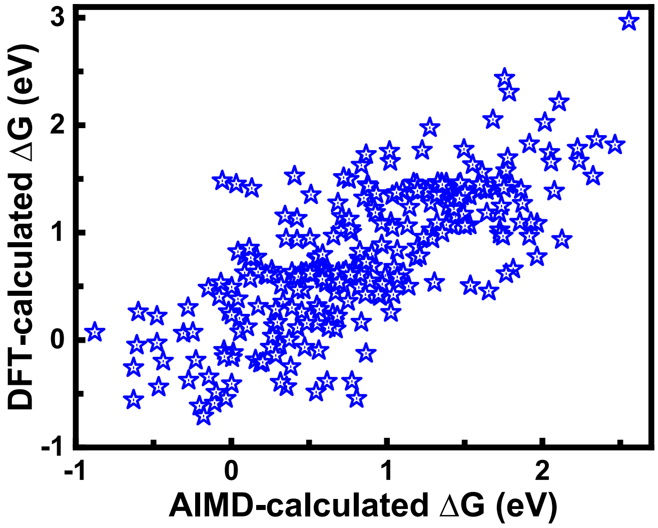

Figure S6. DFT-calculated versus AIMD-calculated Gibbs free energy of intermediates.

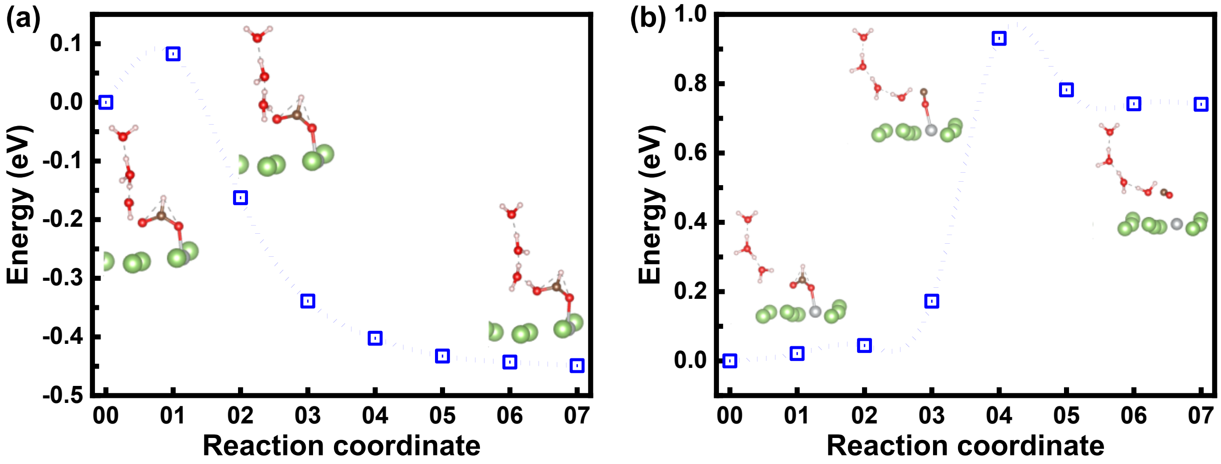

Figure S7. Kinetic investigation. The free energy barrier (CI-NEB calculations) for (a) $CHOO^* \xrightarrow{H^++e^-} CHOOH^*$ and (b) $CHOO^* \xrightarrow{H^++e^-} CO^* + H_2O$. It shows the maximum energy barrier of 0.08 and 0.53 eV for $CHOO^* \xrightarrow{H^++e^-} CHOOH^*$ in the forward and backward directions, respectively. It shows the maximum energy barrier of 0.93 and 0.19 eV for $CHOO^* \xrightarrow{H^++e^-} CO^* + H_2O$ in the forward and backward directions, respectively.

We employed the climbing image nudged elastic band (CI-NEB) method, as implemented in the VTST package, to calculate the energy barriers. The DFT parameters used are consistent with those described in the Materials and Methods section of the manuscript. To account for the explicit

polarization effects involved in proton transfer events, three explicit water molecules were added to the reaction system, and structural relaxation was performed using VASPsol. Each CI-NEB calculation included six intermediate images to accurately resolve the transition states.

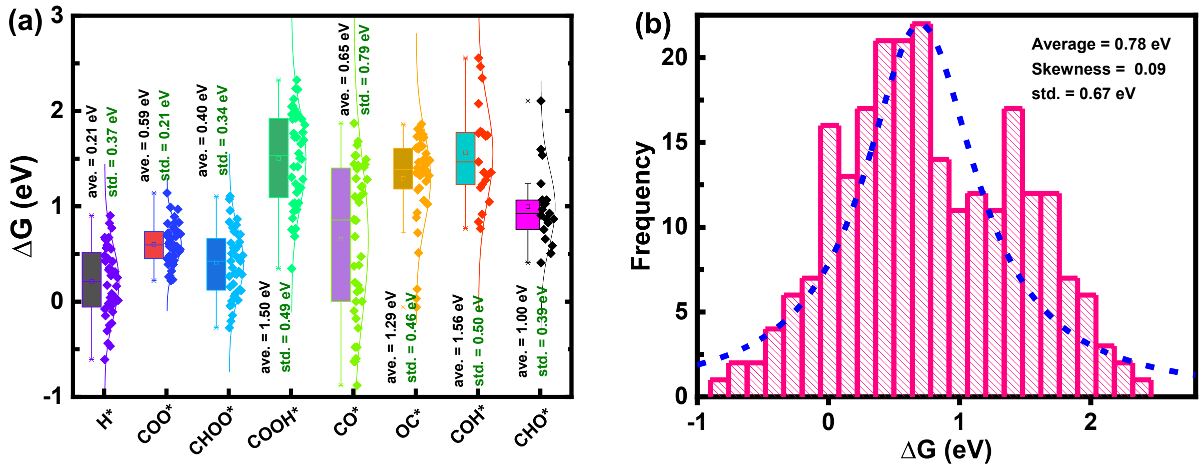

Figure S8. (a) Box plot showing the variation of AIMD-calculated ΔG of reaction intermediates for M-SAC@Ga. (b) The histogram of ΔG variation, calculated from AIMD simulations, contains 240 data points with an average of 0.78 eV, a skewness of 0.09, and a standard deviation (std.) of 0.67 eV.

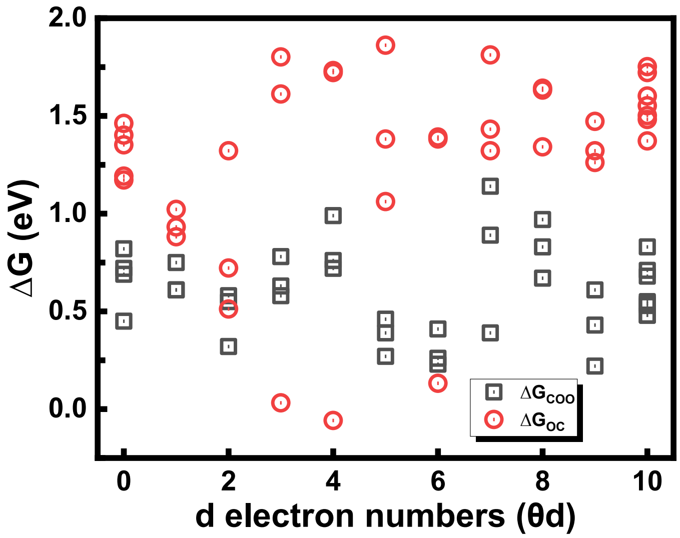

Figure S9. The dependence of the Gibbs free energy of COO and OC intermediates versus the d-electron number of active sites.

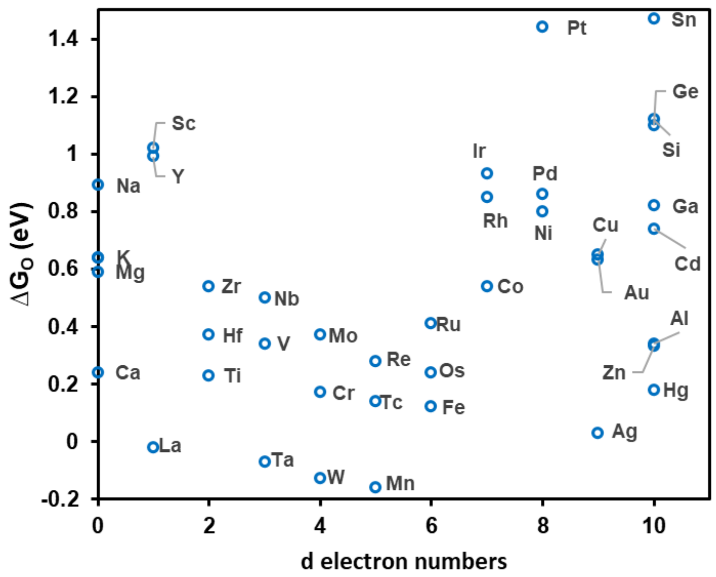

Figure S10. The relationship of Gibbs free energy of oxygen intermediate versus the d-electron number (θ_d) of active sites indicates the strongest adsorption at the d-electron number of $\theta_d = 5$.

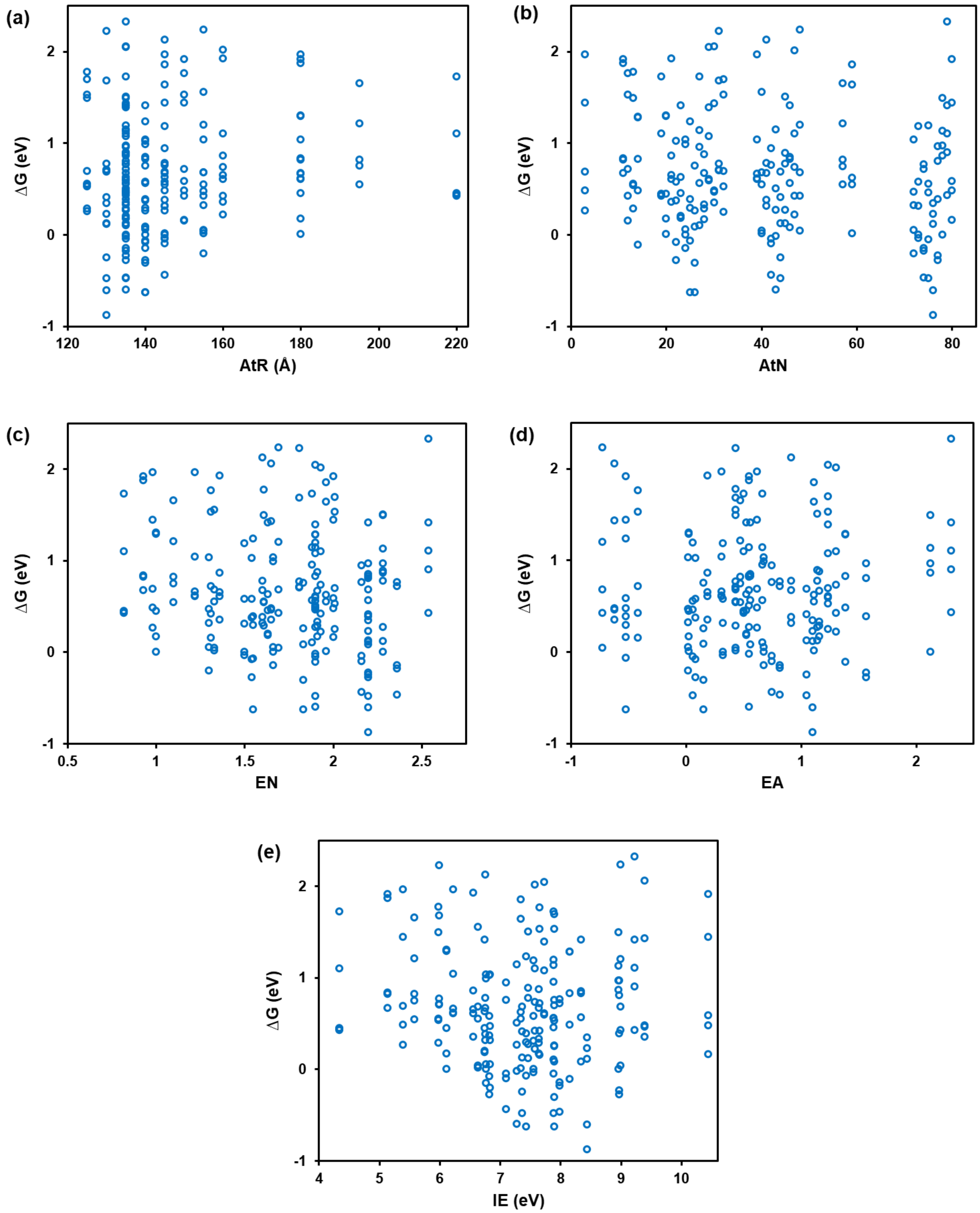

Figure S11. AIMD-calculated Gibbs free energy of reaction intermediates versus (a) atomic radius (AtR), (b) atomic number (AtN), (c) electronegativity (EN), (e) electron affinity (EA), and (d) first ionization energy (IE_M).

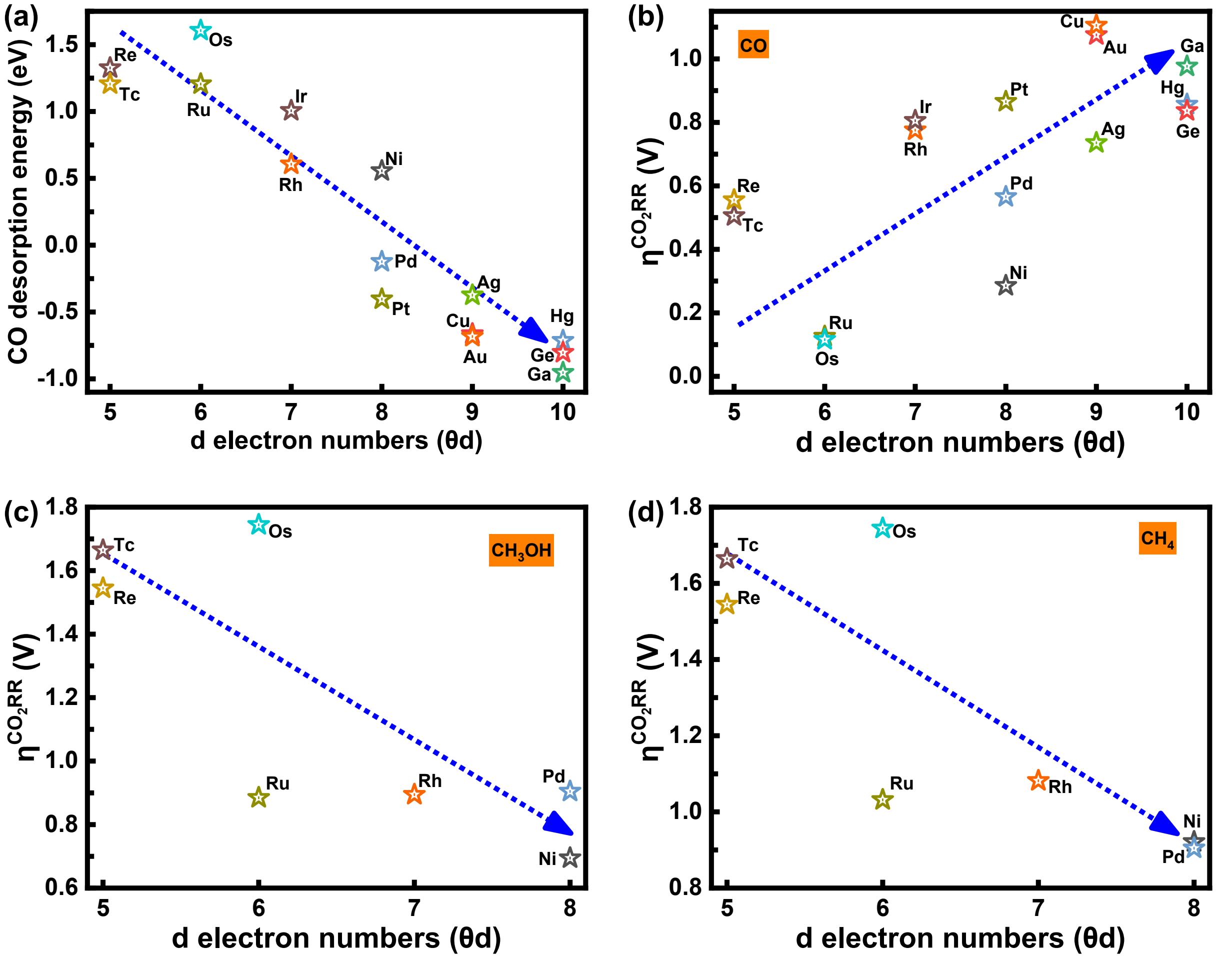

Figure S12. (a) CO desorption energy versus the d-electron number (θ_d) of dopants. The overpotential of CO_2RR toward (a) CO, (c) CH_3OH, and (d) CH_4 versus the d-electron number (θ_d) of dopants.

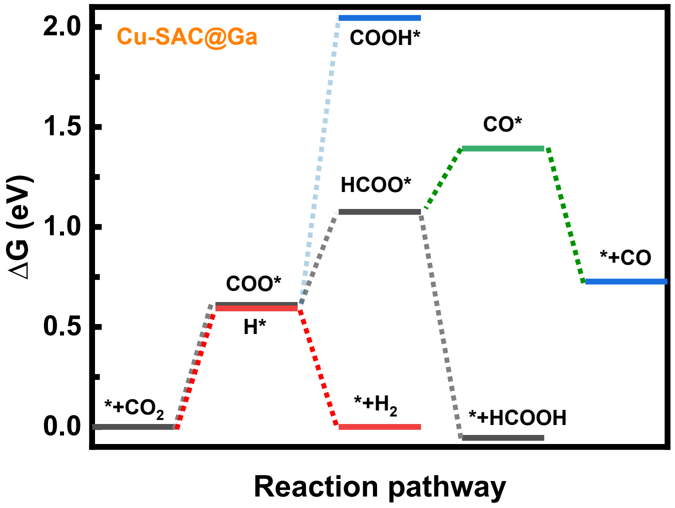

Figure S13. CO_2RR pathway for Cu-SAC@Ga.

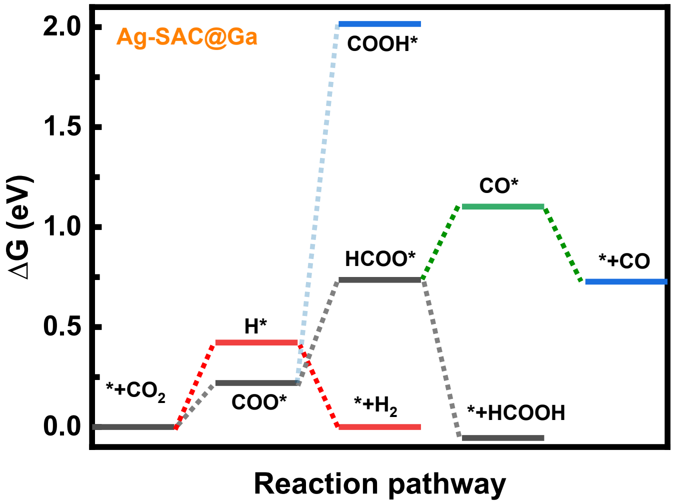

Figure S14. CO_2RR pathway for Ag-SAC@Ga.

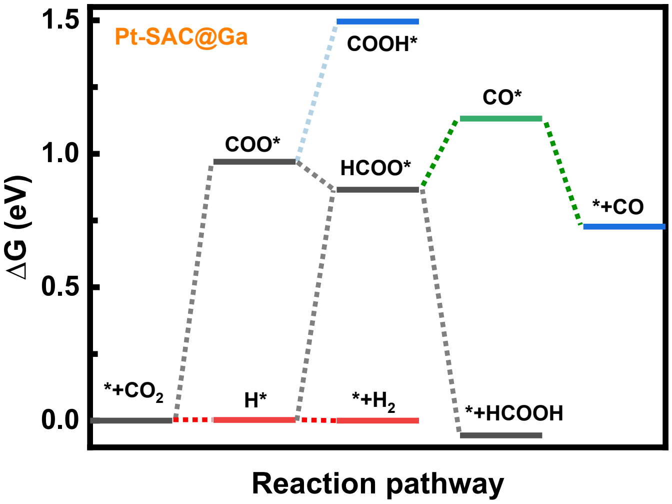

Figure S15. CO_2RR pathway for Pt-SAC@Ga.

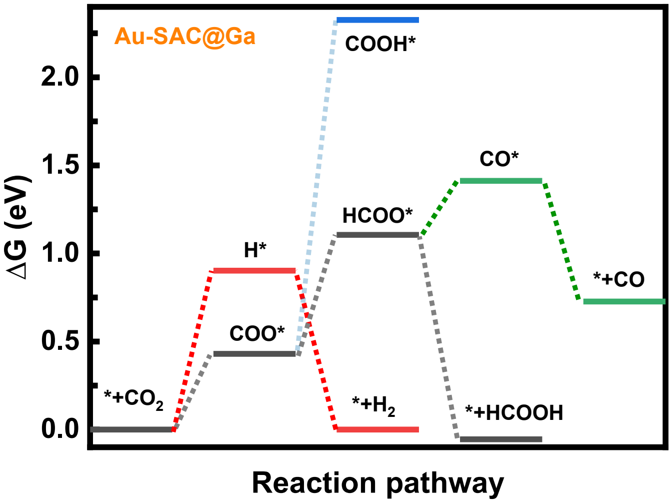

Figure S16. CO_2RR pathway for Au-SAC@Ga.

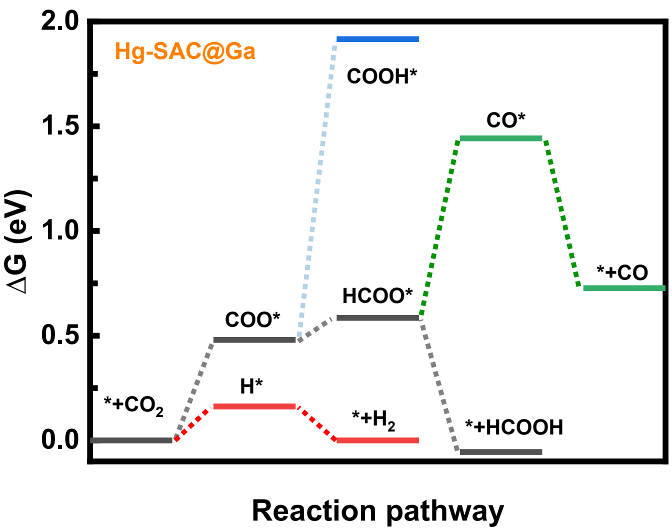

Figure S17. CO_2RR pathway for Hg-SAC@Ga.

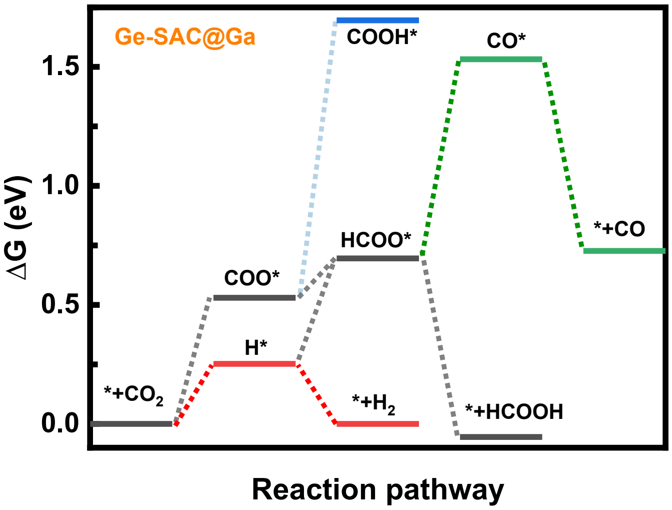

Figure S18. CO_2RR pathway for Ge-SAC@Ga.

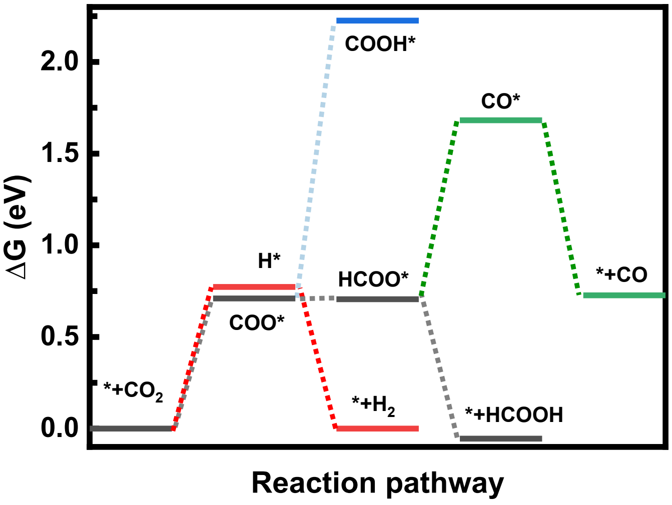

Figure S19. CO_2RR pathway for undoped Gallium.

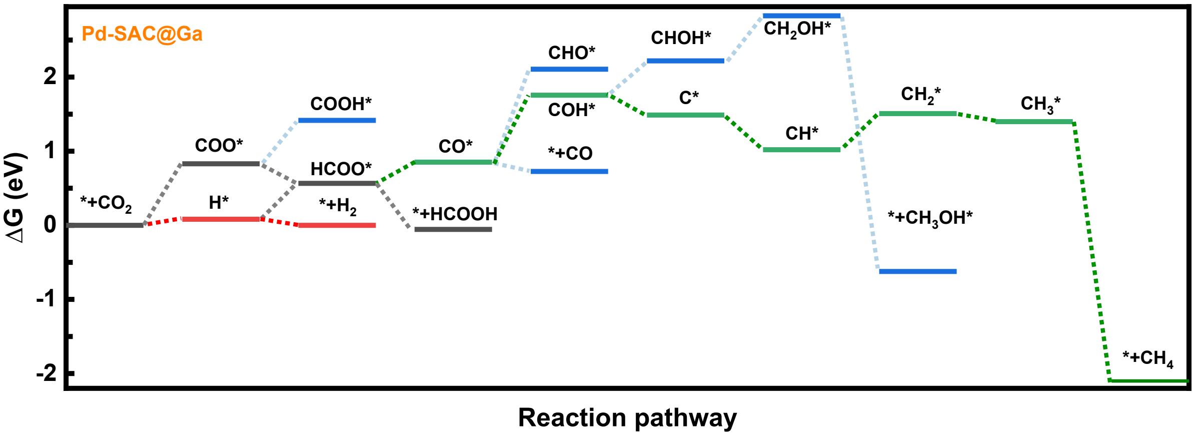

Figure S20. CO_2RR pathway for Pd-SAC@Ga.

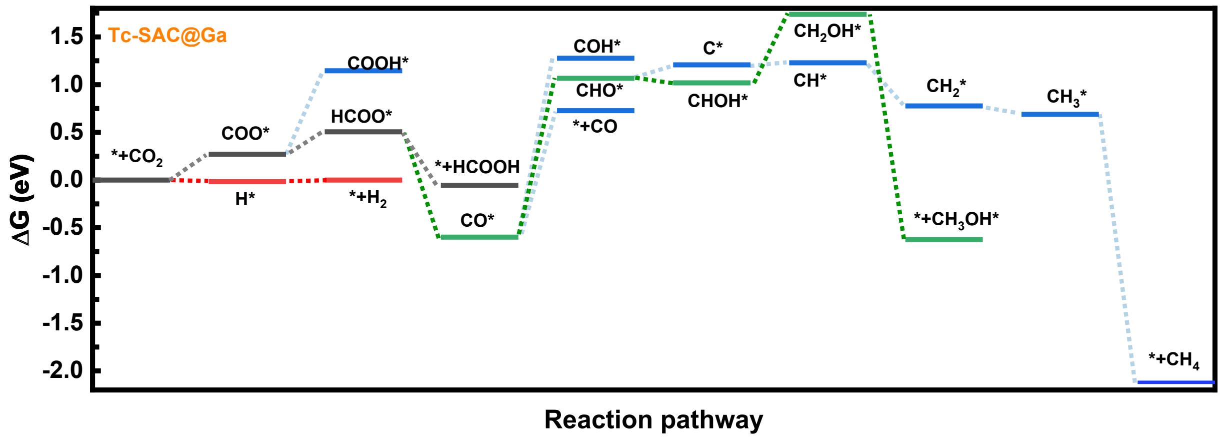

Figure S21. CO_2RR pathway for Tc-SAC@Ga.

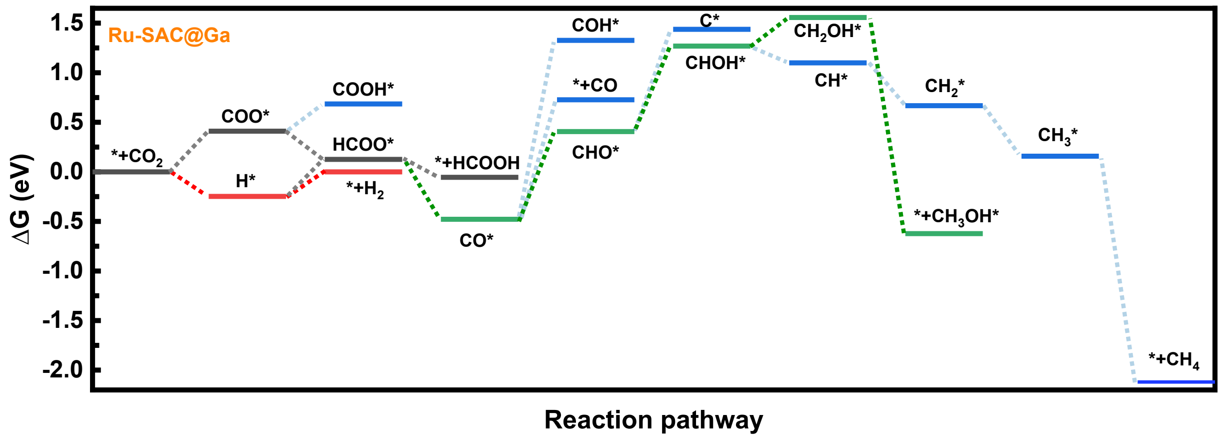

Figure S22. CO_2RR pathway for Ru-SAC@Ga.

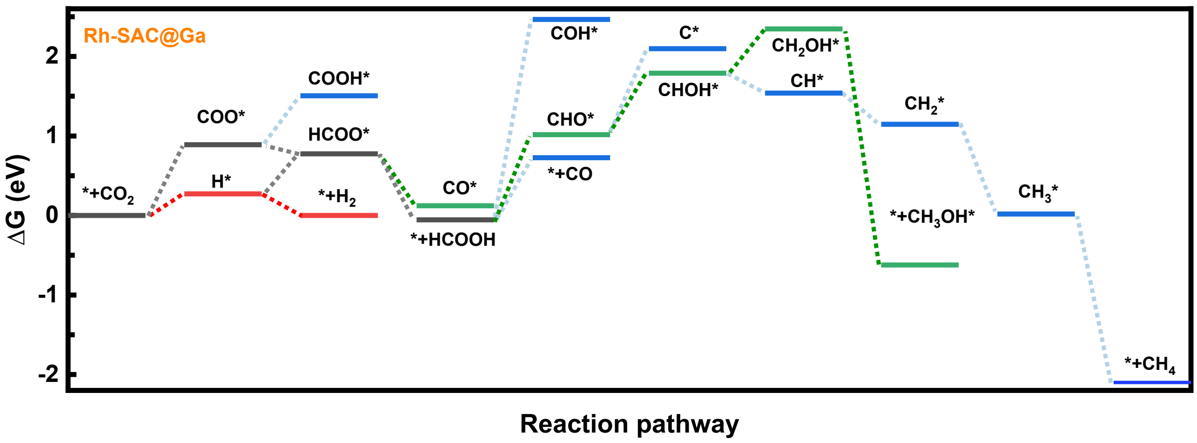

Figure S23. CO_2RR pathway for Rh-SAC@Ga.

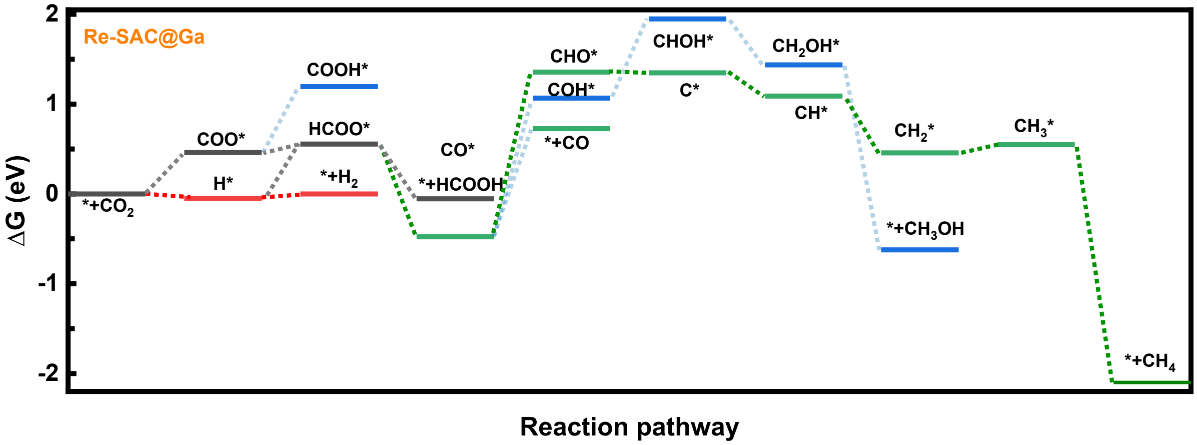

Figure S24. CO_2RR pathway for Re-SAC@Ga.

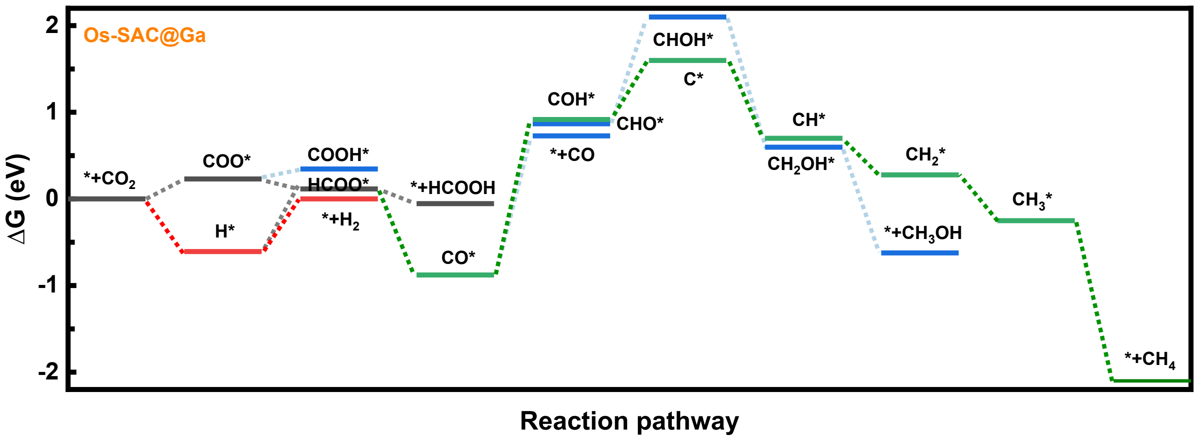

Figure S25. CO_2RR pathway for Os-SAC@Ga.

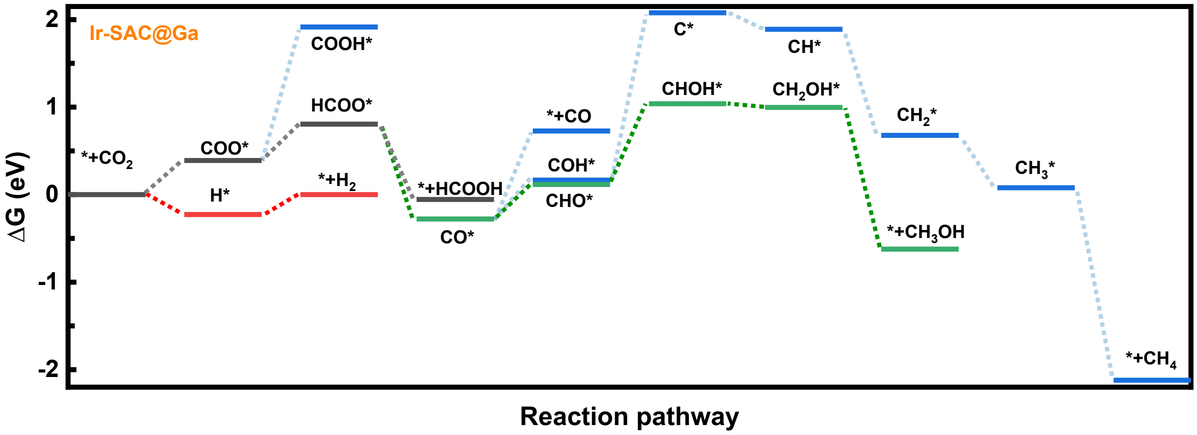

Figure S26. CO_2RR pathway for Ir-SAC@Ga.

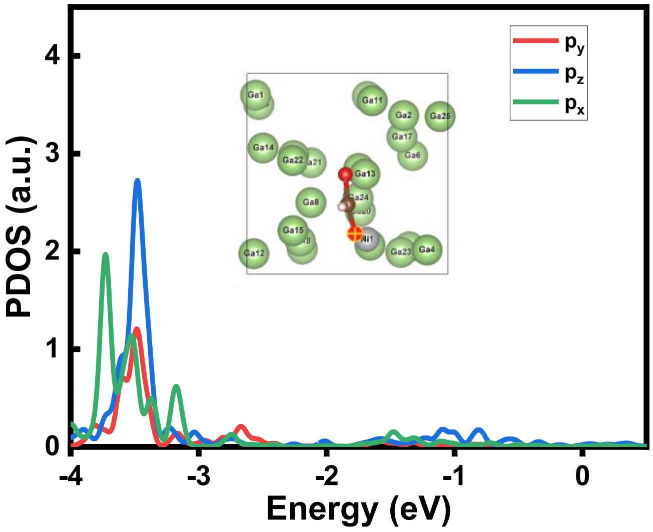

Figure S27. Partial density of states (PDOS) of p_y, p_z, and p_x orbitals of the O atom in CHOO intermediate adsorbed on Ni-SAC@Ga.

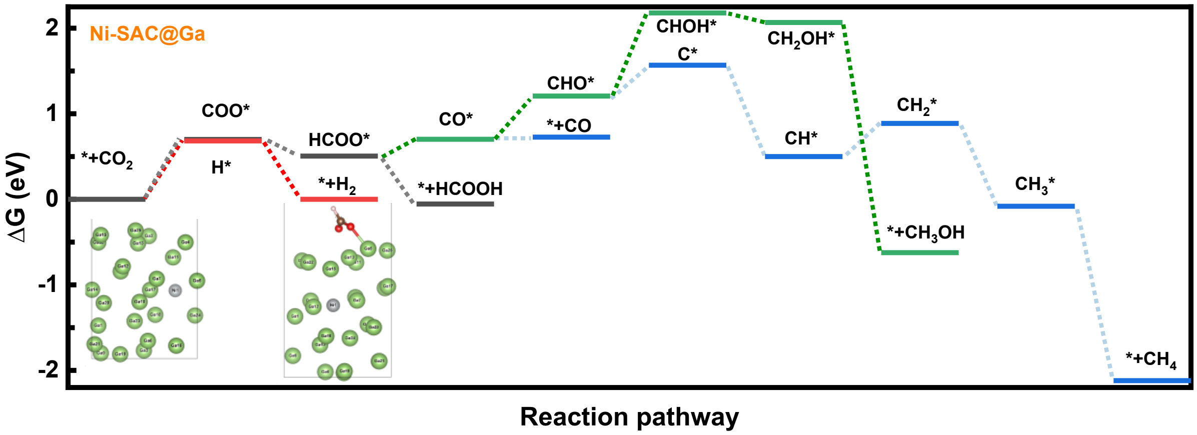

Figure S28. CO_2RR pathway for Ni-SAC@Ga (where the Ni atom is embedded within the bulk of Ga).

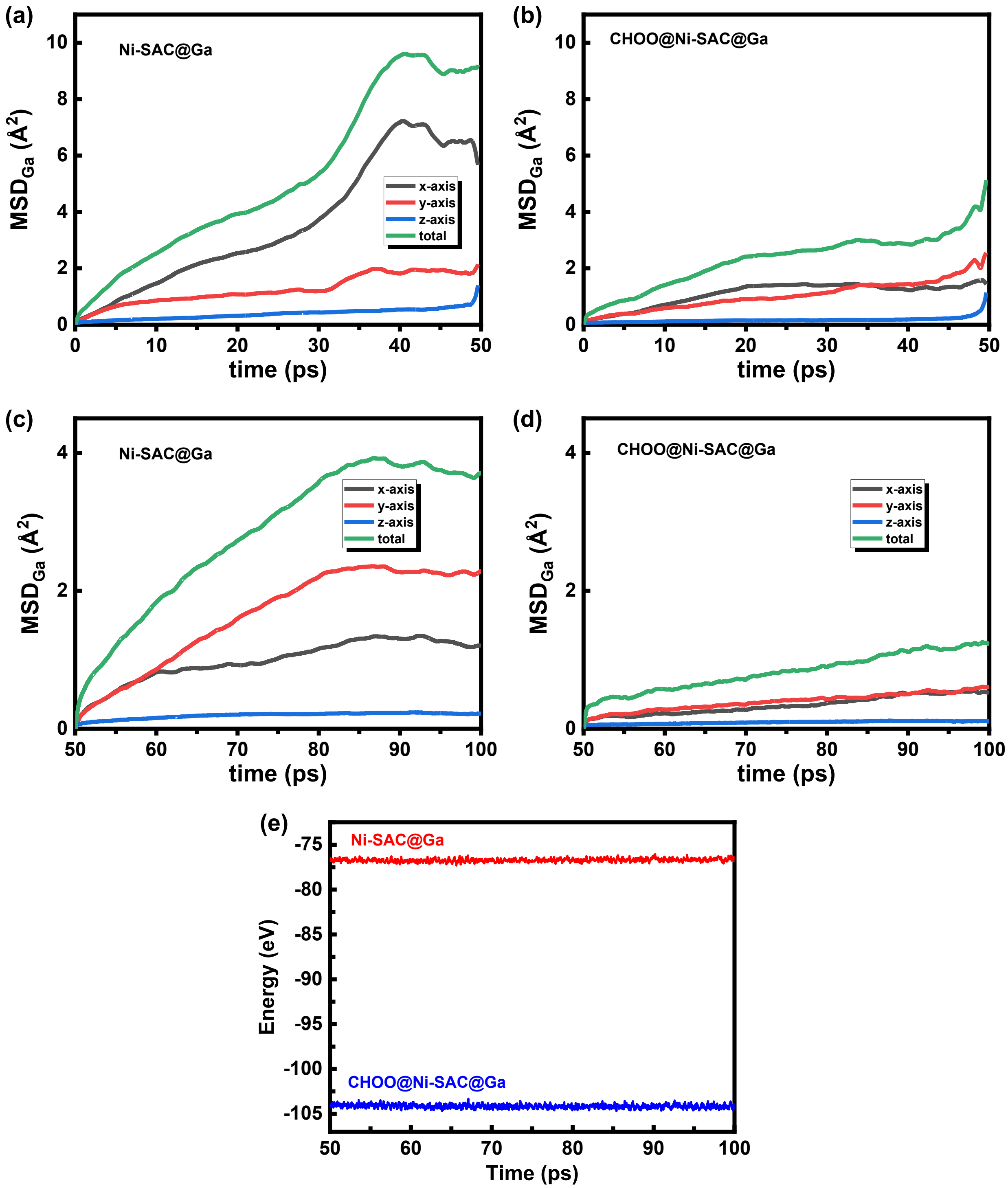

Figure S29. Mean squared displacement (MSD) of Ga atoms in the Ni-SAC@Ga system over a 0-50 ps window (a) in the absence and (b) presence of the CHOO intermediate. MSD of Ga atoms in the Ni-SAC@Ga system over a 50-100 ps window (c) in the absence and (d) presence of the CHOO intermediate. (e) Total energy profiles of Ni-SAC@Ga and CHOO@Ni-SAC@Ga over the 50-100 ps simulation window.

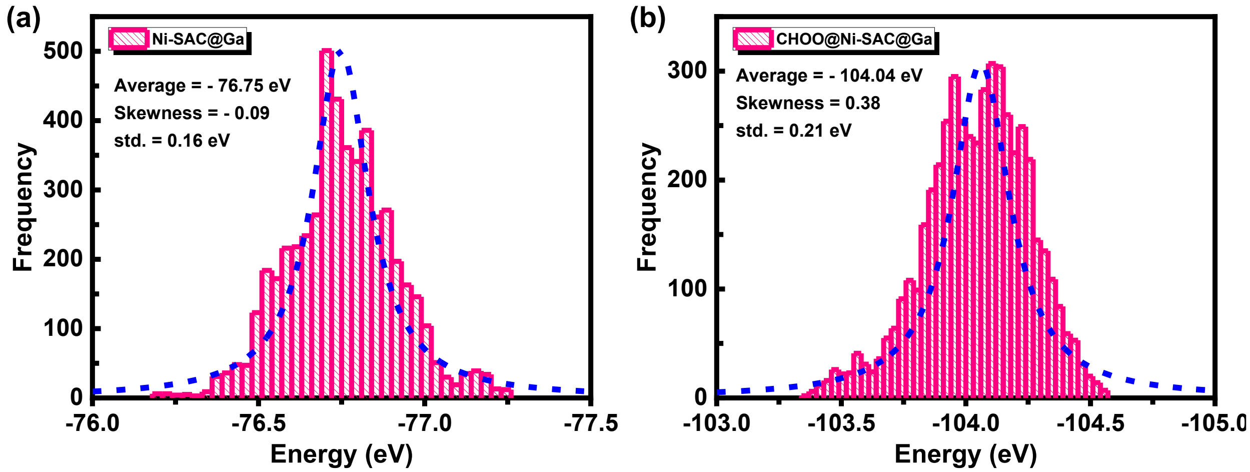

Figure S30. The histogram of the total energy of (a) Ni-SAC@Ga and (b) CHOO@Ni-SAC@Ga obtained from AIMD-D3 calculations, indicating the average, standard deviation, and skewness of the energies from 40 to 50 picoseconds, with a bin size of 0.03 eV.

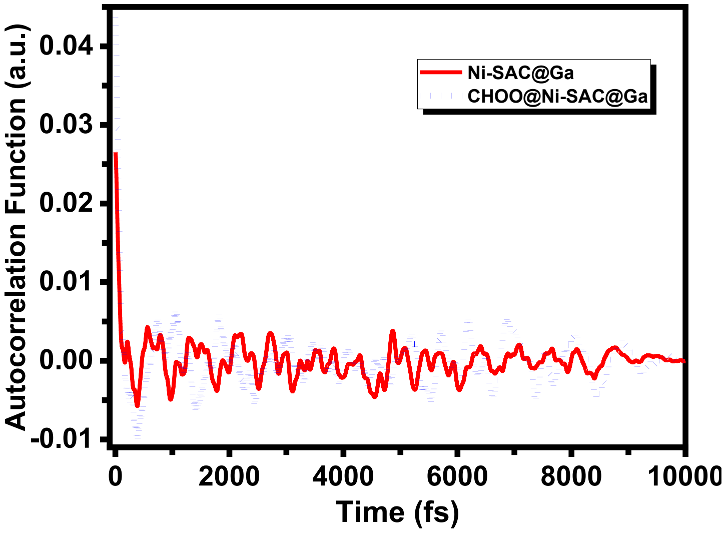

Figure S31. Autocorrelation function in Ni-SAC@Ga and CHOO@Ni-SAC@Ga, related to the total energy at 300 K from 40 to 50 picoseconds.

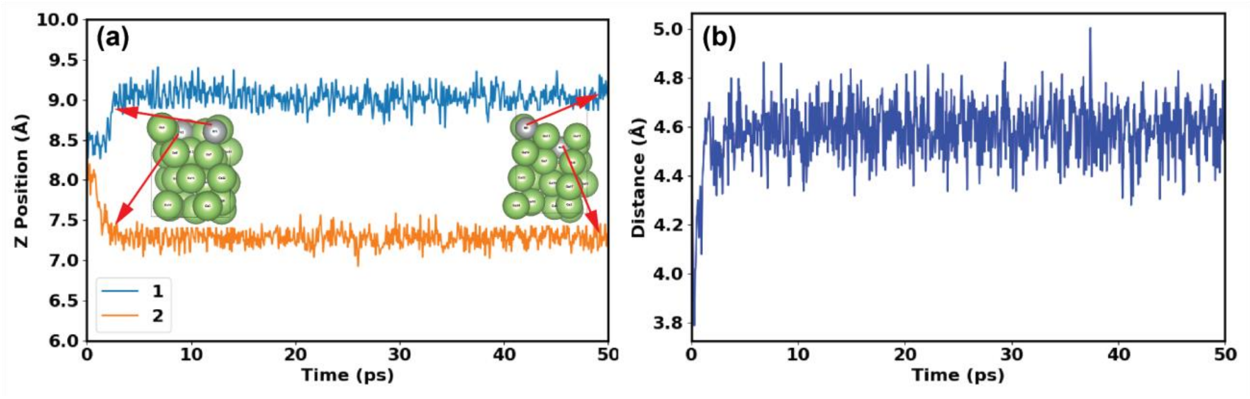

Figure S32. (a) Z-position of Ni atoms in Ni_2-SAC@Ga obtained from AIMD simulations at 300 K (26.85 °C). The insets illustrate the atomic structure of Ni and Ga atoms. The surface of the catalysts is at ≃ 9 Å. (b) Distance between two Ni atoms in Ni_2-SAC@Ga obtained from AIMD simulations at 300 K (26.85 °C).

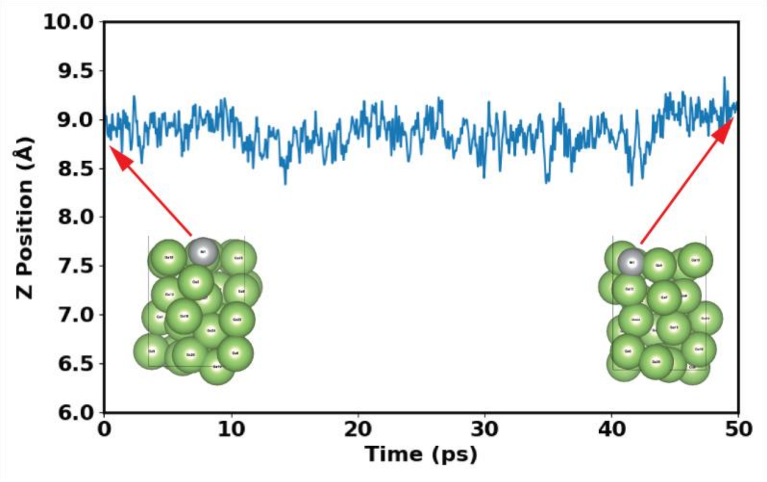

Figure S33. Z-position of the Ni atom in Ni-SAC@Ga obtained from AIMD simulations at 300 K (26.85 °C). The insets illustrate the atomic structure of Ni and Ga atoms. The surface of the catalysts is at ≃ 9 Å.

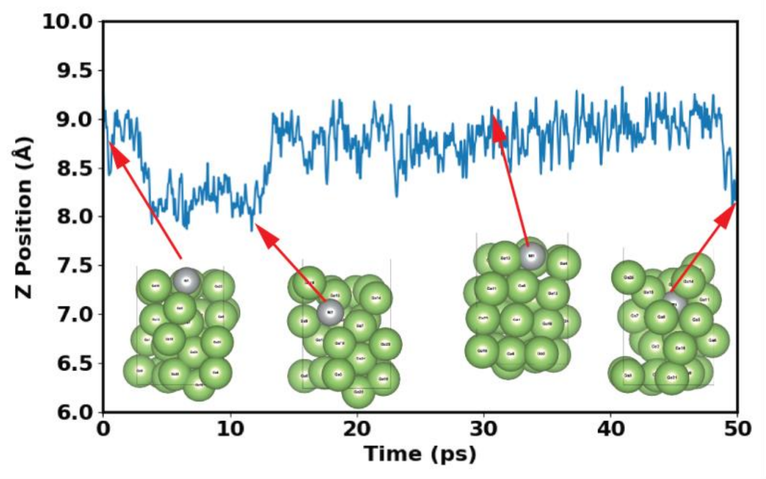

Figure S34. Z-position of the Ni atom in Ni-SAC@Ga obtained from AIMD simulations at 673.15 K (400 ºC). The insets illustrate the atomic structure of Ni and Ga atoms. The surface of the catalysts is at ≃ 9 Å.

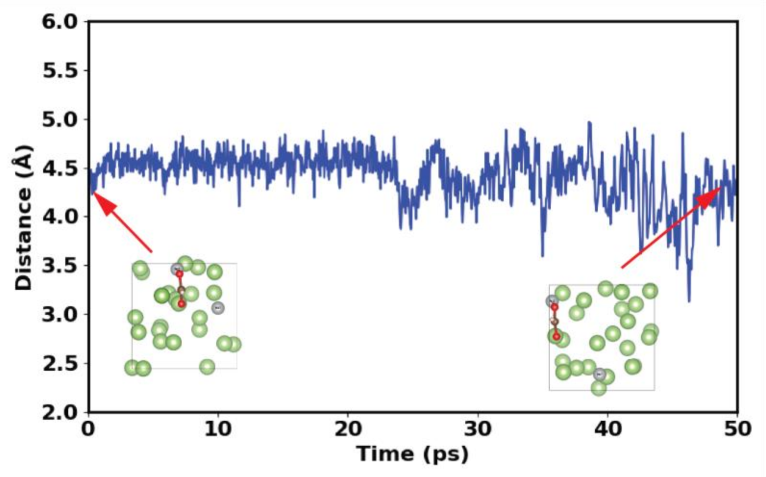

Figure S35. Distance between two Ni atoms in Ni_2-SAC@Ga in the presence of CHOO intermediate, obtained from AIMD simulations at 300 K (26.85 °C).

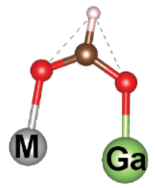

Figure S36. The structure of the CHOO intermediate on M-SAC@Ga.

CONTCAR file of CHOO@Ni-SAC@Ga after 50 picoseconds AIMD simulation.

```
CHOO@Ni-SAC@Ga
  1.00000000000000
    7.9099998469999999    0.0000000000000000    0.0000000000000000
    0.0000000000000000    7.9099998469999999    0.0000000000000000
    0.0000000000000000    0.0000000000000000   20.7639999390000014
  Ga   Ni    C    O    H
  25    1    1    2    1
Direct
  0.0426009890333843  0.8917380413087431  0.2043874886680784
  0.7796682874614733  0.7902127196639129  0.2951280641642694
  0.2750164417857451  0.1247360758793972  0.0623697759366744
  0.8957895394045456  0.1201920221549106  0.4551213632289952
  0.6118267581565751  0.1439601831824793  0.3223984847380709
  0.8241109832559939  0.5905598104317179  0.0379170682466554
  0.5561381340792509  0.5269251681980804  0.2536247541897899
  0.3178946824294381  0.3561826763530150  0.3058476250443693
  0.5991524130137256  0.8856405336738216  0.0132346097537681
  0.2316359659811663  0.5907274555170665  0.1734520120325820
  0.6232198090989712  0.8640694437358414  0.4335532416848294
  0.0338690344868588  0.1017063015617858  0.3271574424120324
  0.5862225566009731  0.4979733285941961  0.4238059911103235
  0.0796910746655453  0.6286686701026650  0.2933448248184711
  0.2285205231524286  0.2161644965815034  0.4339931821336526
  0.8068013272288749  0.1360068510878529  0.0774272151389606
  0.7710766250895134  0.6834539129066474  0.1653568028343703
  0.2649606882325687  0.1645943926524752  0.2021528556411825
  0.0585872373027725  0.8452989072562846  0.0505800188650866
  0.5636441728478323  0.3120450431653584  0.0005602488479360
  0.3192741836214958  0.5546216966104766  0.0452986512149036
  0.2266296967379257  0.5659838512173712  0.4170608970987088
  0.7654386792377611  0.1083113458215417  0.2066727266777655
  0.5523736450571254  0.3793658132679508  0.1359669956128695
  0.9607486627174991  0.7854736341960342  0.4240297683045211
  0.5973507867139468  0.1726562972151488  0.4356221682137775
  0.5008662882490665  0.3471146442448174  0.5529985821148592
  0.5372357842204537  0.2017572957653276  0.5279800897506556
  0.4896868432164003  0.4967136841069064  0.5293003880169812
  0.4726656020869407  0.3351821431914658  0.6048166140339890
```

S2. Machine Learning and Data Analysis

We use XGBoost as a supervised machine learning (ML) algorithm to predict the Gibbs free energy of different intermediates using data from the AIMD calculations. We used the XGBoost, Scikit-learn, NumPy, Matplotlib, Pickle, and SciPy libraries in Python 3.6 to read and process the data, train and save the ML algorithm, and perform feature importance analysis. In order to have high-quality data analysis and an interpretable ML algorithm, we need physically interpretable input features with high simplicity and reasonable feature importance.[6] Accordingly, the input features used in our ML model include the intrinsic properties of element (M), substrate, and intermediates such as atomic radius (AtR_M), atomic number (AtN_M), electronegativity (EN_M), first ionization energy (IE_M), electron affinity (EA_M), numbers of electron in d orbitals of the active site ($|\theta_d\text{-}6|$), and valence electron number of reaction intermediate (int_{VE1}, int_{VE2}, and int_{VE3}). The input dataset contains a total of 200 AIMD-predicted Gibbs free energies of H, COO, COOH, CHOO, and CO. The input data was randomly partitioned into the training set (90%, 180 data points) and the test set (10%, 20 data points). The training set was used to construct the ML model, and we used the root mean squared error (RMSE, equation S1) and R^2 value to evaluate the performance of the ML model:[7]

$$\mathrm{RMSE} = \sqrt{\frac{1}{n}\sum_{i=1}^{n}\left(\Delta G_{AIMD,i} - \Delta G_{ML,i}\right)^2} \qquad \text{(S15)}$$

where $\Delta G_{AIMD,i}$ and $\Delta G_{ML,i}$ are the AIMD- and ML-calculated Gibbs free energies, respectively, for intermediate i, and n is the number of instances in the training data set. Based on the trained XGBoost model, several methods such as Shapley Additive exPlanation (SHAP),[8] permutation,[9] mutual information (MI), and the Pearson correlation coefficient[10] were applied to screen the impact of each input feature on the model output.

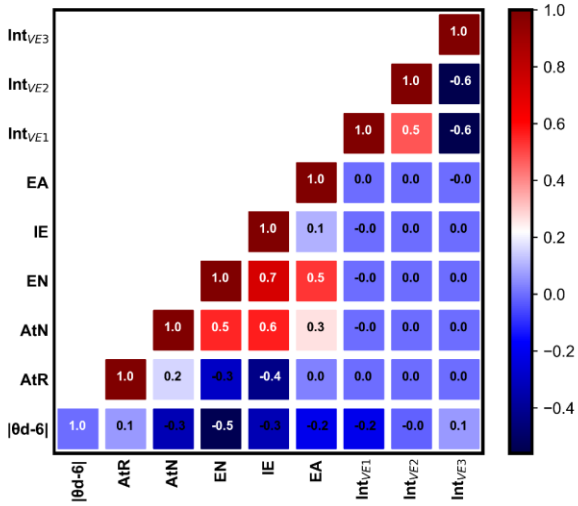

Figure S37. Feature-feature Pearson correlation map of the input features.

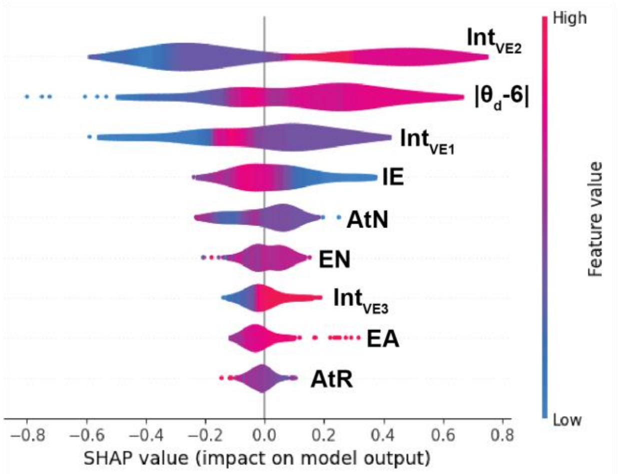

Figure S38. Violin plot of SHAP values for input features colored based on the value of the feature.

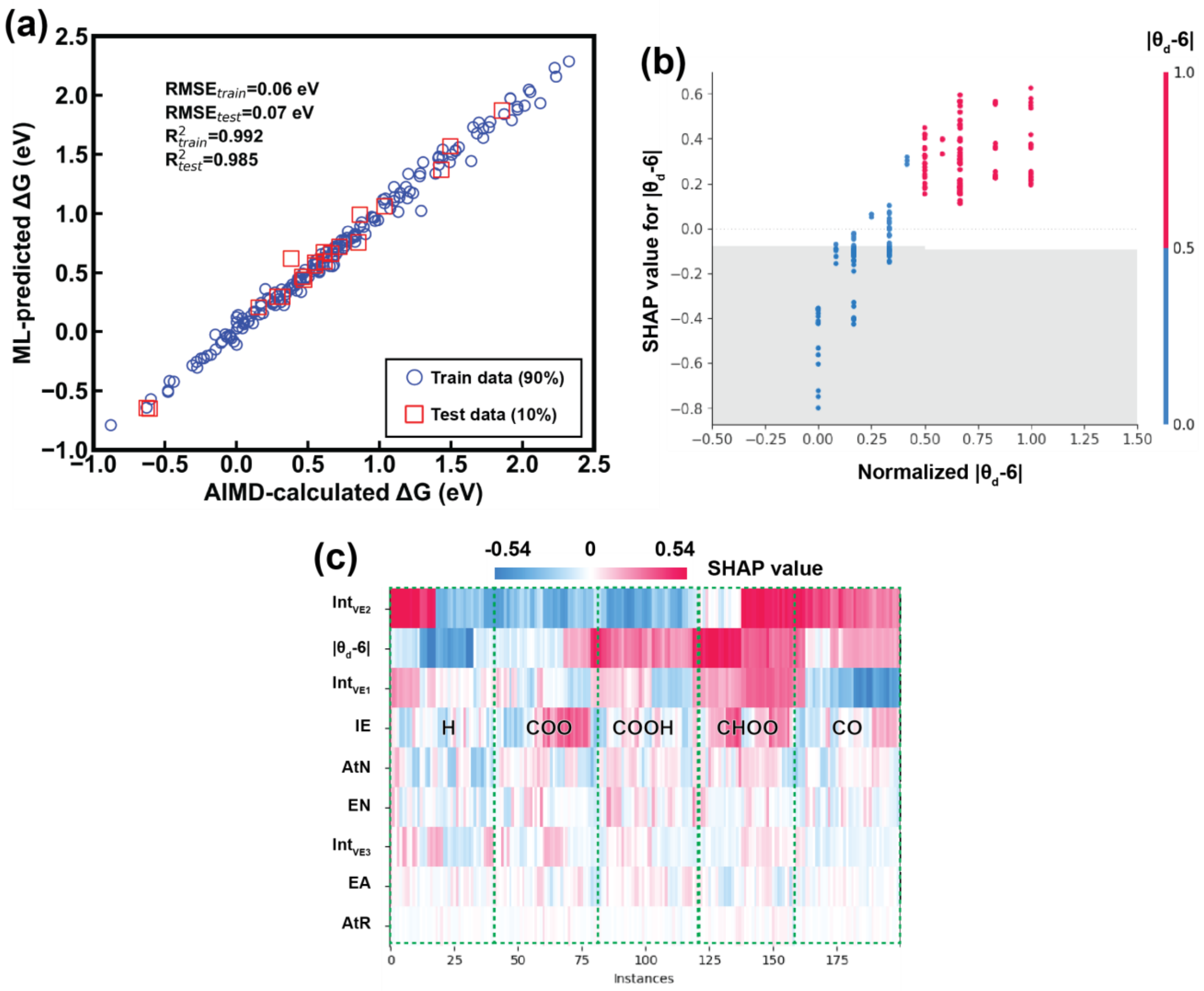

Figure S39. Machine learning implementation. (a) The parity plot of ML-predicted versus AIMD-calculated Gibbs free energy of reaction intermediates such as H, COO, COOH, CHOO, and CO for M-SAC@Ga. The XGBoost algorithm shows satisfactory MSE and R^2 values for training and test data without any signs of underfitting. (b) SHAP value of $|\theta_d$-6| versus normalized $|\theta_d$-6|, colored based on the features' values. (c) The heatmap of SHAP values of the input features for the whole 200 data instances in the order of feature importance.

S3. References

(1) Rehman, F.; Kwon, S.; Musgrave, C. B.; Tamtaji, M.; Goddard III, W. A.; Luo, Z. High-Throughput Screening to Predict Highly Active Dual-Atom Catalysts for Electrocatalytic Reduction of Nitrate to Ammonia. *Nano Energy* **2022**, *103*, 107866. https://doi.org/10.1016/j.nanoen.2022.107866.

(2) CRC Handbook of Chemistry and Physics. *CRC Handb. Chem. Phys.* **2016**. https://doi.org/10.1201/9781315380476.

(3) Lide, D. R. *CRC Handbook of Chemistry and Physics*; CRC press, 2004; Vol. 85.

(4) Nolan, B. M.; Chan, E. K.; Zhang, X.; Muthuswamy, E.; Van Benthem, K.; Kauzlarich, S. M. Sacrificial Silver Nanoparticles: Reducing GeI2 to Form Hollow Germanium Nanoparticles by Electroless Deposition. *ACS Nano* **2016**, *10* (5), 5391–5397. https://doi.org/10.1021/acsnano.6b01604.

(5) Firdaus, M.; Andriana, S.; Elvinawati; Alwi, W.; Swistoro, E.; Ruyani, A.; Sundaryono, A. Green Synthesis of Silver Nanoparticles Using Carica Papaya Fruit Extract under Sunlight Irradiation and Their Colorimetric Detection of Mercury Ions. *J. Phys. Conf. Ser.* **2017**, *817* (1), 012029. https://doi.org/10.1088/1742-6596/817/1/012029.

(6) Tamtaji, M.; Gao, H.; Hossain, M. D.; Galligan, P. R.; Wong, H.; Liu, Z.; Liu, H.; Cai, Y.; Goddard, W. A.; Luo, Z. Machine Learning for Design Principles for Single Atom Catalysts towards Electrochemical Reactions. *J. Mater. Chem. A* **2022**, *10* (29), 15309–15331. https://doi.org/10.1039/d2ta02039d.

(7) Tamtaji, M.; Guo, X.; Tyagi, A.; Galligan, P. R.; Liu, Z.; Roxas, A.; Liu, H.; Cai, Y.; Wong, H.; Zeng, L.; Xie, J.; Du, Y.; Hu, Z.; Lu, D.; Goddard III, W. A.; Zhu, Y.; Luo, Z. Machine Learning-Aided Design of Gold Core-Shell Nanocatalysts toward Enhanced and Selective Photooxygenation. *ACS Appl. Mater. Interfaces* **2022**, *14* (41), 46471–46480. https://doi.org/10.1021/acsami.2c11101.

(8) Anker, A. S.; Kjaer, E. T. S.; Juelsholt, M.; Christiansen, T. L.; Linn Skjaervø, S.; Ry, M.; Jørgensen, V.; Kantor, I.; Sørensen, D. R.; Billinge, S. J. L.; Selvan, R.; Jensen, K. M. Ø. Extracting Structural Motifs from Pair Distribution Function Data of Nanostructures

Using Explainable Machine Learning. *npj Comput. Mater.* **2022**, *8* (1), 3–5. https://doi.org/10.1038/s41524-022-00896-3.

(9) Mi, X.; Zou, B.; Zou, F.; Hu, J. Permutation-Based Identification of Important Biomarkers for Complex Diseases via Machine Learning Models. *Nat. Commun.* **2021**, *12* (1), 3008. https://doi.org/10.1038/s41467-021-22756-2.

(10) Panapitiya, G.; Avendano-Franco, G.; Ren, P.; Wen, X.; Li, Y.; Lewis, J. P. Machine-Learning Prediction of CO Adsorption in Thiolated, Ag-Alloyed Au Nanoclusters. *J. Am. Chem. Soc.* **2018**, *140* (50), 17508–17514. https://doi.org/10.1021/jacs.8b08800.
